# Supplementary material for: Hierarchical crystal structure prediction of zeolitic imidazolate frameworks using DFT and machine-learned interatomic potentials
Source: Chem Sci. 2026 Sep 4. Online ahead of print. doi: 10.1039/d6sc04307k (PMC13584093; doi:10.1039/d6sc04307k)
Supplement: Supplementary file 1 [file SC-OLF-D6SC04307K-s001.pdf]

Supplementary Information (SI) for Chemical Science.
This journal is © The Royal Society of Chemistry 2026

Electronic Supplementary Information

Hierarchical Crystal Structure Prediction of Zeolitic Imidazolate Frameworks Using DFT and Machine-Learned Interatomic Potentials

Yizhi Xu,[a,b] Jordan Dorrell,[c,d] Katarina Lisac,[b] Ivana Brekalo,[b] James P. Darby,[e] Andrew J. Morris[c]* and Mihails Arhangelskis [a]*

[a]Faculty of Chemistry, University of Warsaw; 1 Pasteura Street, Warsaw 02-093, Poland.

[b]Division of Physical Chemistry, Ruđer Bošković Institute, Zagreb, Croatia

[c]School of Metallurgy and Materials, University of Birmingham, Edgbaston, Birmingham B15 2TT, U.K.

[d]School of Chemistry and Chemical Engineering, University of Southampton, Southampton, SO17 1BJ, UK

[e]Symmetric Group, 71-75 Shelton Street, Covent Garden, London, WC2H 9JQ, UK

Corresponding authors:

*Prof. Andrew J. Morris, E-mail: a.j.morris@bham.ac.uk

*Dr. Mihails Arhangelskis, E-mail: m.arhangelskis@uw.edu.pl

Table of Contents

S1. COMPUTATIONAL METHODS 3

S1.1 Structure generation by Wyckoff Alignment of Molecules (WAM) method 3
S1.2 Periodic DFT calculations 3
S1.3 Training of machine-learned potentials 3
S1.4 Geometry optimization and energy ranking using machine learned potentials 12
S1.5 Post-processing of the predicted crystal structures 12
S1.6 Protocol for comparing experimental PXRD patterns with predicted CSP structures via Critic2 13
S1.7 Periodic DFT optimization of the structures deemed likely experimental candidates 13

S2. THE USE OF SEPARATE MLIPS FOR ENERGIES AND FORCES 14

S3. MECHANOCHEMICAL SYNTHESIS 16

S4. RIETVELD REFINEMENT 16

S5. COMPARISON OF DFT AND ML ENERGIES FOR THE PREDICTED STRUCTURES 17

S6. DISTRIBUTION OF FRAMEWORK TOPOLOGIES WITHIN THE ENERGY LANDSCAPE 19

S7. SCALING OF RELATIVE ENERGIES WITH RESPECT TO CALCULATED VOID FRACTION. 32

S8. COMPARISON OF THE PREDICTED STRUCTURES AGAINST EXPERIMENTAL POWDER DIFFRACTION PATTERNS. 34

S9. REFERENCES 39

S1. Computational methods

S1.1 Structure generation by Wyckoff Alignment of Molecules (WAM) method

Crystal structures were generated using AIRSS[1]+WAM[2], separately for 1, 2, 3, 4, 6, 8, 10, 12, 14 and 16 formula units of Zn(**Im**)$_2$ per primitive unit cell, with each formula unit containing one Zn atom and two imidazolate fragments. In each search, space groups with up to 8 symmetry operations were uniformly sampled. Zn atoms and imidazolate linker fragments were placed at random positions within the trial cell, subject to space group symmetry constraints. In addition, minimal separation constraints were set to prevent overlap of different atomic fragments in the trial configurations. The total number of structures generated for each number of formula units is shown in Table S1.

Table S1. The number of structures generated for each number of Zn(**Im**)$_2$ formula units.

Number of formula units per primitive cell	Number of generated structures
1	500
2	1000
3	1500
4	3000
6	72047
8	295714
10	694635
12	743386
14	703491
16	1222749

S1.2 Periodic DFT calculations

All putative randomly generated Zn(**Im**)$_2$ structures by WAM, containing 1 to 4 formula units per primitive cell were geometry optimized with the plane-wave periodic density-functional theory (DFT) code CASETP19.[3] The calculations were performed using LDA functional and the plane-wave cut-off was set to 400 eV. The ultrasoft pseudopotentials from the CASTEP internal library were used, while the first Brillion zone was sampled with a 2π x 0.07 Å^{-1} Monkhorst Pack k-point grid.[4] Structures were optimized with respect to both lattice parameters and atomic positions, while enforcing the symmetry constraints defined by the WAM-assigned space group. The convergence criteria for the geometry optimizations were set to be maximum energy change of 2x10^{-5} eV atom^{-1}, maximum force on atom of 0.05 eV Å^{-1}, maximum atom displacement of 0.001 Å and residual stress of 0.1 GPa.

S1.3 Training of machine-learned potentials

Herein, the deep neural network-based software SchNetPack 1.0.0[5] (GitHub commit 6fe78ef), enabling the polarizable interaction neural network (PaiNN) architecture,[6] was employed to train the machine-learned interatomic potentials (MLIP). For the training, all 6000 aforementioned periodic DFT-optimized Zn(**Im**)$_2$ structures, containing 1 to 4 formula units per primitive cell, were utilized. The preparation of training data was performed via our internal code, ML-Tools. Specifically, geometry optimization snapshots, including

unit cell parameters, atomic positions and forces, were extracted from individual .castep geometry optimization files, converted into an ASE[7] atoms object, and stored in a database format compatible with SchNetPack. The starting and final geometry configurations were always included, while the intermittent steps were sampled according to a two-part process. Initially, we discarded most steps, retaining only every 20th step. Next, we randomly sampled the remaining steps, where the probability of a step being stored was weighted by the fractional distance through a geometry optimization walk according to Gaussian distribution, drawing more structures from the initial geometry steps, where the differences between successive energy steps are larger, ensuring greatest data diversity. We also ensured that no single geometry optimization trajectory is shared between training, validation and sets. In total, 19311 structural snapshots were extracted from the CASTEP geometry optimization output files, with the training, validation and test set split being 10972, 2739 and 5600, respectively. Separate MLIPs were constructed for energies and forces, the reasons for training separate potentials rather than a single one are discussed in section S2.

The hyperparameters for training the energies and forces MLIP were all set in the configuration .yaml file within the SchNetPack software. The cutoff distance was set to 5 Å, where the software would consider as pairwise atoms as neighbours with non-zero interaction energy. Number of radial basis functions were set to 20, whereas number of atomic basis sets of 128 was used. For the number of interaction blocks, the value of 3 was used. Batch size was kept at 10 to maintain moderate computational cost. By default, the Adam optimizer[8] was used in training in SchNetPack, and the starting learning rate was set to 5×10^{-4}. The energy key was set to be "formation_energy_per_atom", and "avg" aggregation mode was used to ensure the energy property was treated as a single value per atom. The mean squared error (MSE) loss was set as the loss function. For training the energy and force MLIPs, the loss weight, which is a float number ranging from 0 to 1, was used to regulate the relative contribution of energies and forces to the loss function. For training the energy MLIP, loss weight was set to 1, whereas a loss weight value of 0 was used for the force MLIP.

Detailed analysis of the energy and force distributions in the training, validation and test sets and statistical analysis of predicted errors with the MLIP are shown in Figures S1 - S8.

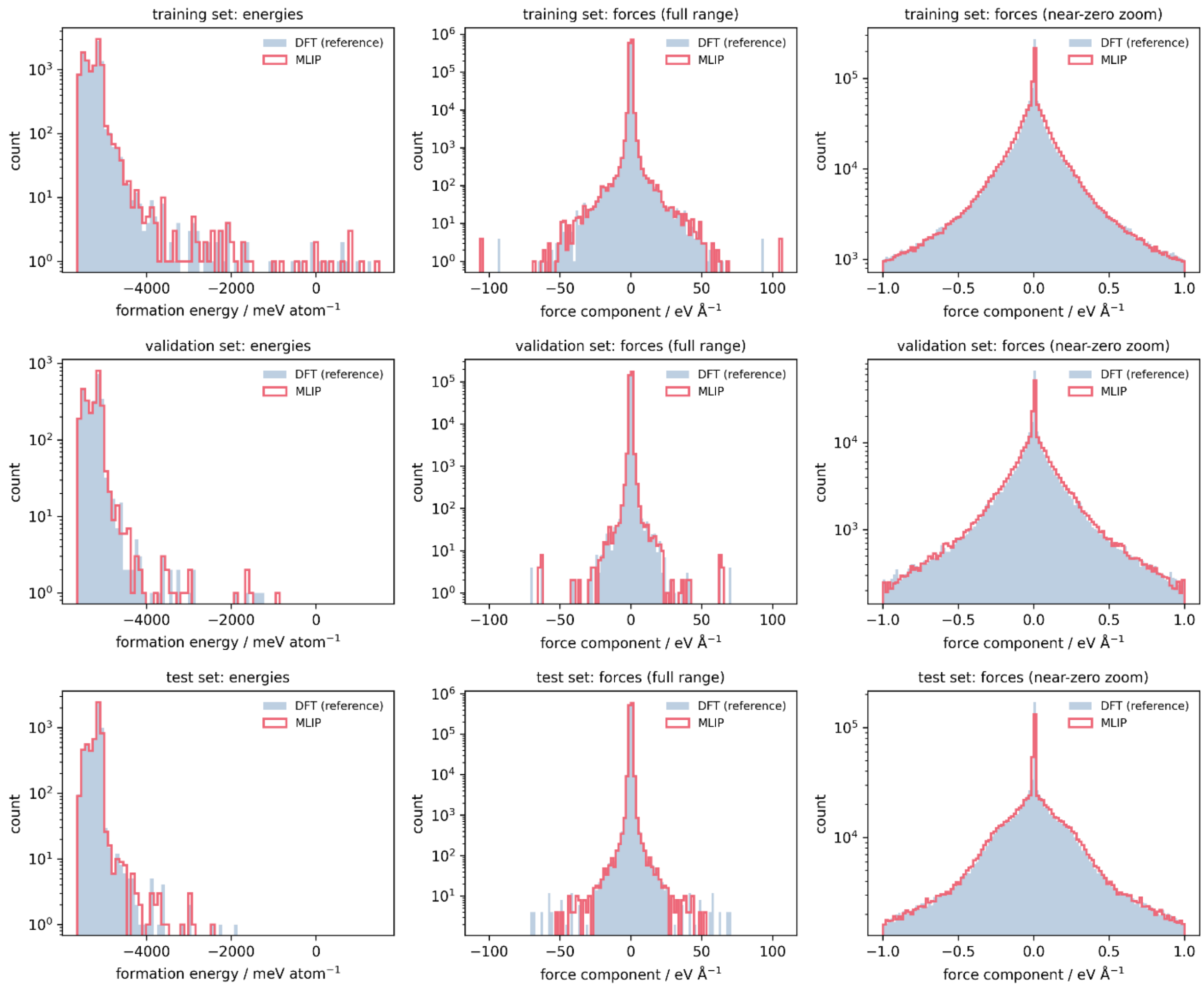

Figure S1. Overlay of distributions for reference (DFT, grey) and MLIP-predicted values (red contour). The two distributions coincide on every panel of every split, including the near-zero force region (right column), which is particularly important for reliable optimization close to the local minima. The forces span a large range of ±110 eV/Å, demonstrating that the training data covers structures far away from equilibrium geometries.

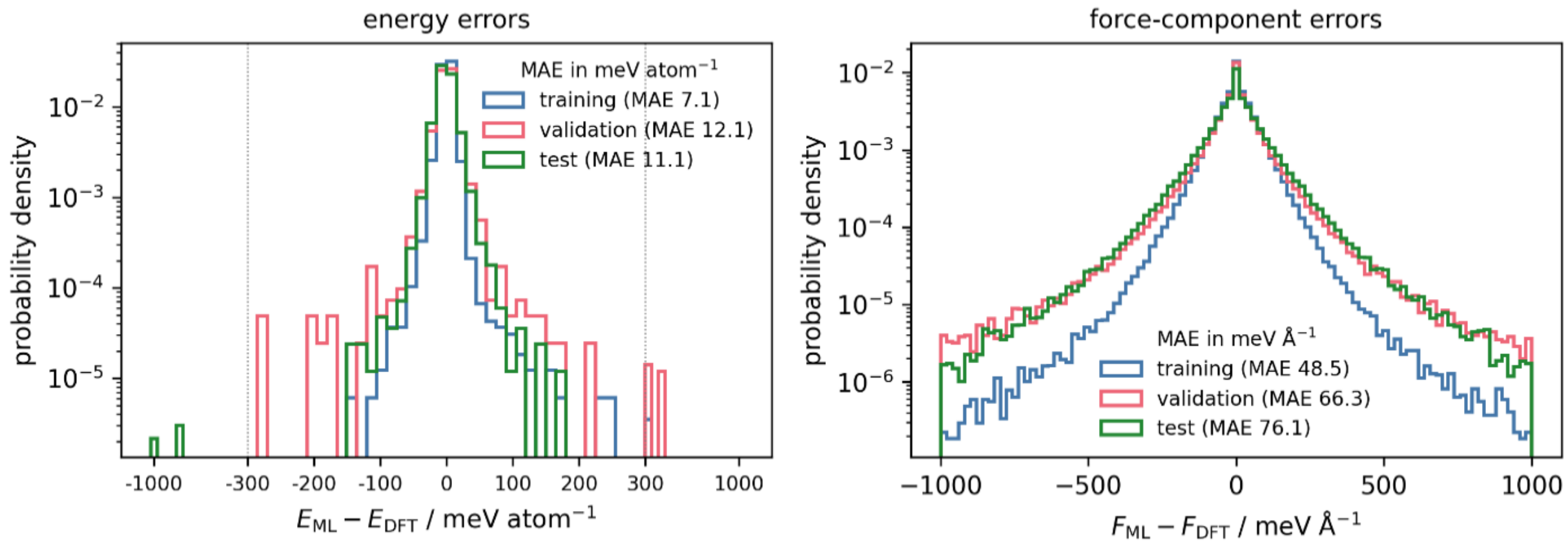

Figure S2. Distributions of the energy and force errors for all three dataset splits are shown. The validation and test distributions are very similar to each other and indicate very mild overfitting compared to the training set errors. MAE = mean absolute error

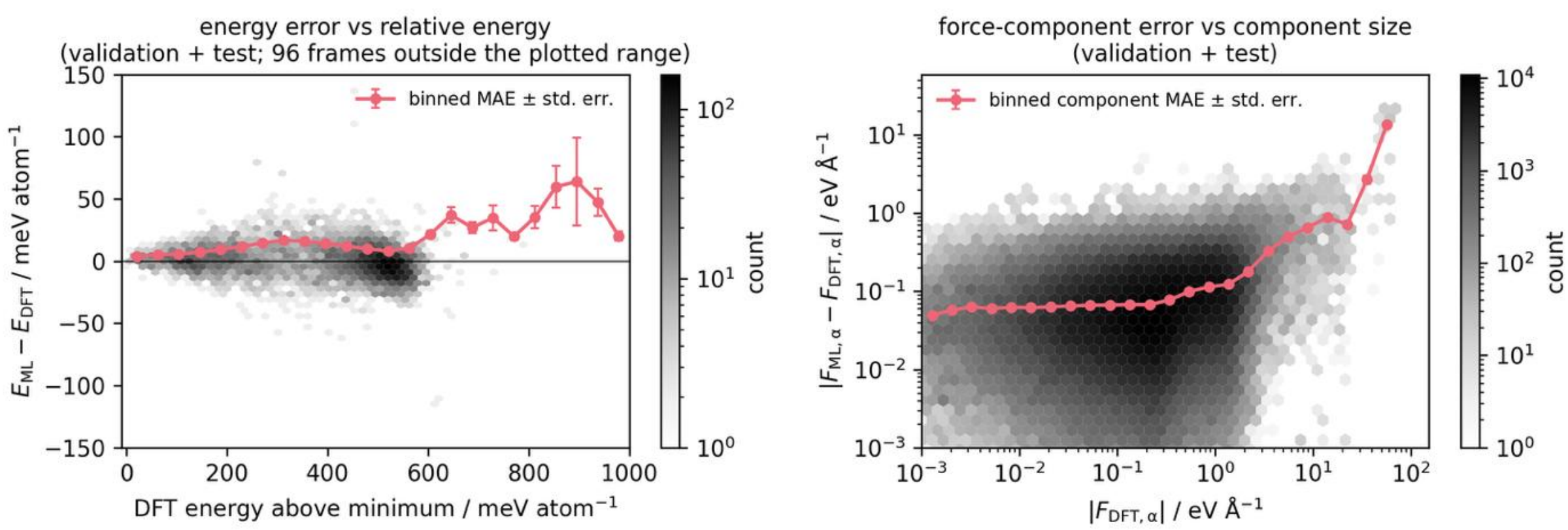

Figure S3. The distribution of energy and force prediction errors as function of formation energy and magnitude of the force components are shown. As expected, the errors are lowest for low energy structures and those with small forces and increase above the average MAE for high energy structures and those far from equilibrium. MAE = mean absolute error

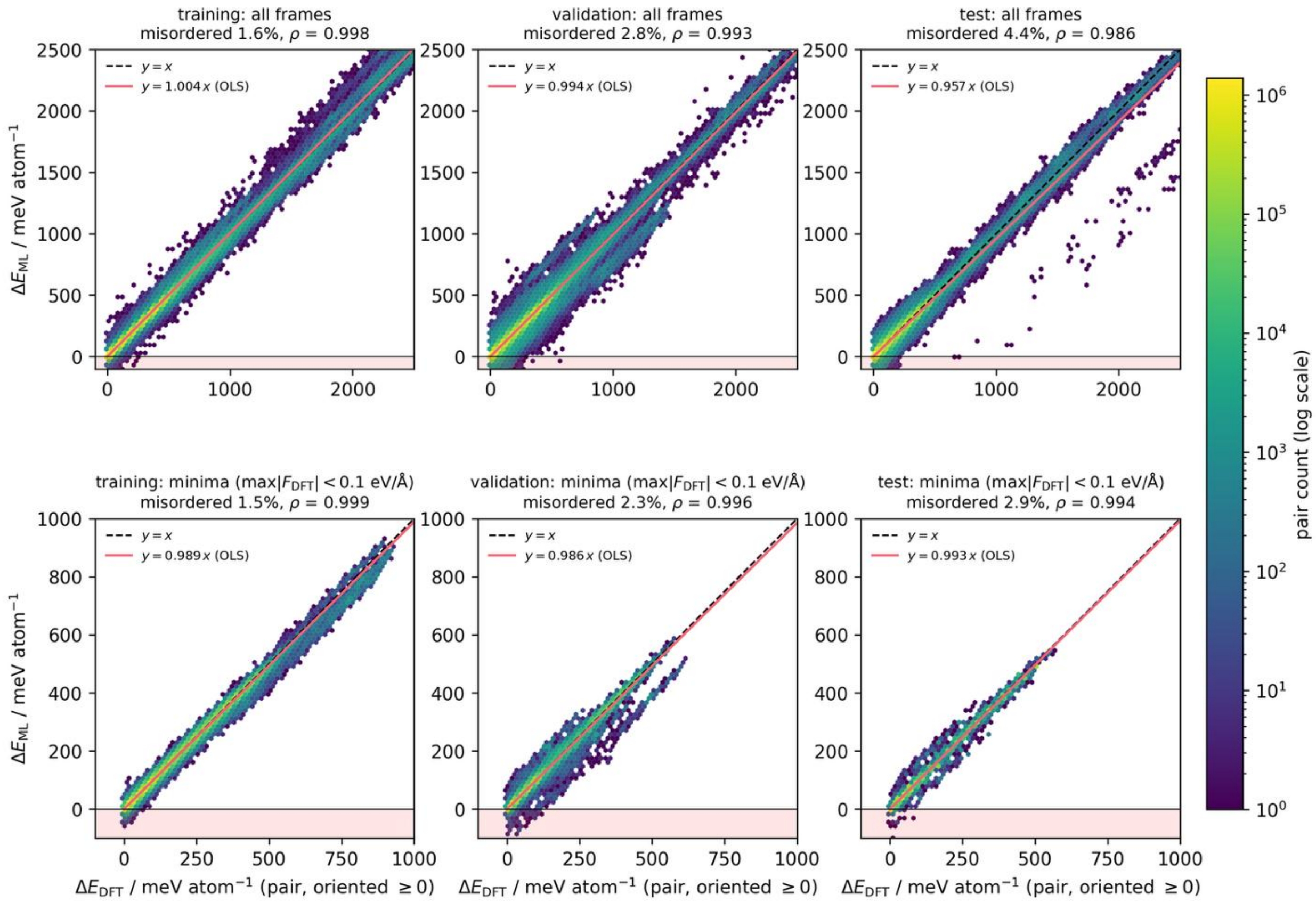

Figure S4. The plots show the parity (MLIP vs DFT, LDA) of pairwise relative energies ΔEij for pairs of structures in the training, validation and test sets, and separately for near-minimum structures (maximum force component of 0.1 eV/ Å) in each set. The pairs have been ordered so that ΔEij > 0 for DFT, so that any incorrect ordering by the MLIP appears in the region marked in red, with the fraction of misordered pairs marked on each plot. The gradients are close to 1 in all six panels – with LDA (which the model was trained on) there is no energy-scale compression. The deviation of the gradient from unity observed in Figure S11 is therefore attributed to comparing against the PBE functional with D3 dispersion correction.

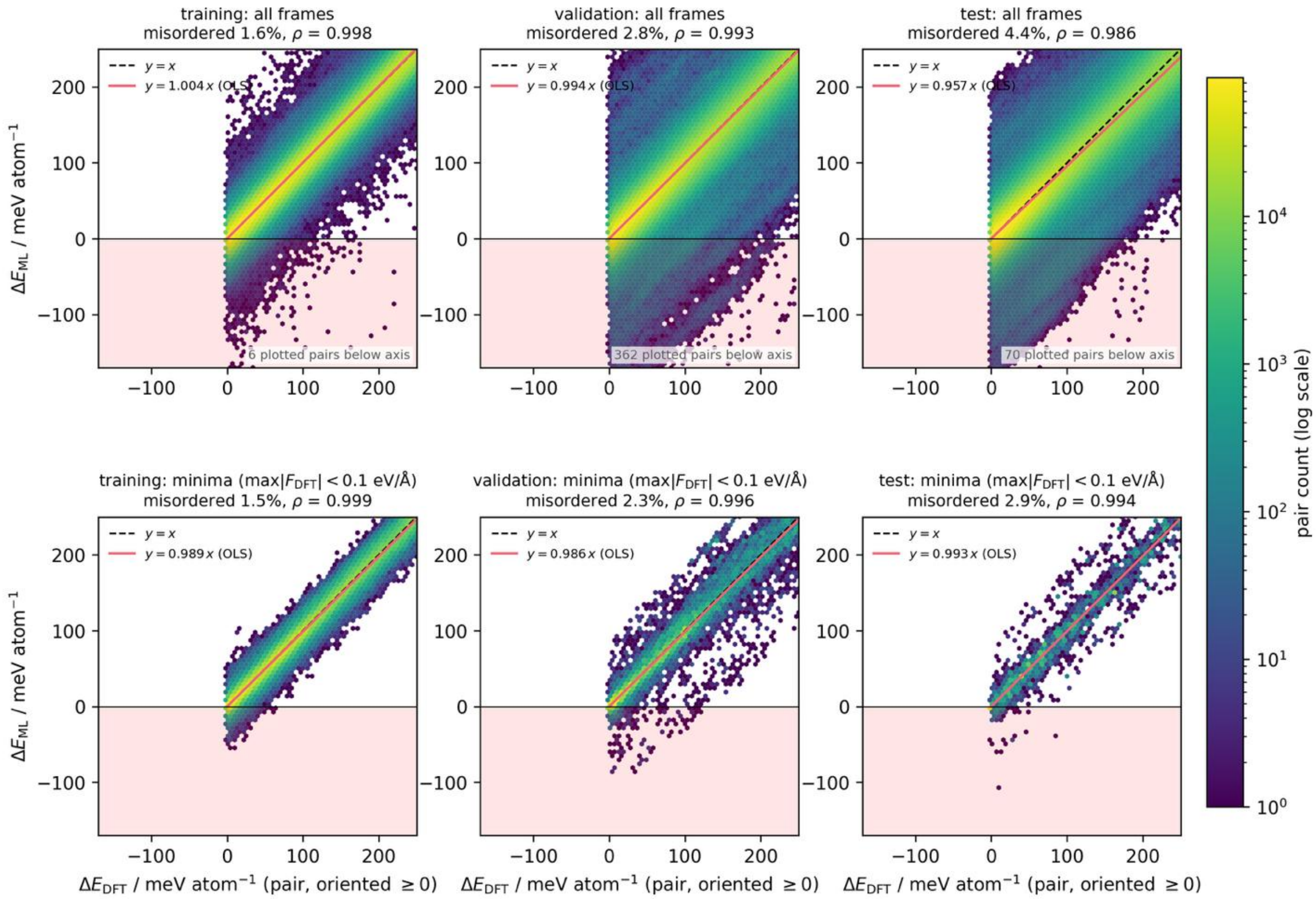

Figure S5. The magnified version of the plots from figure S4, focusing on energies from -100 to +200 meV/atom.

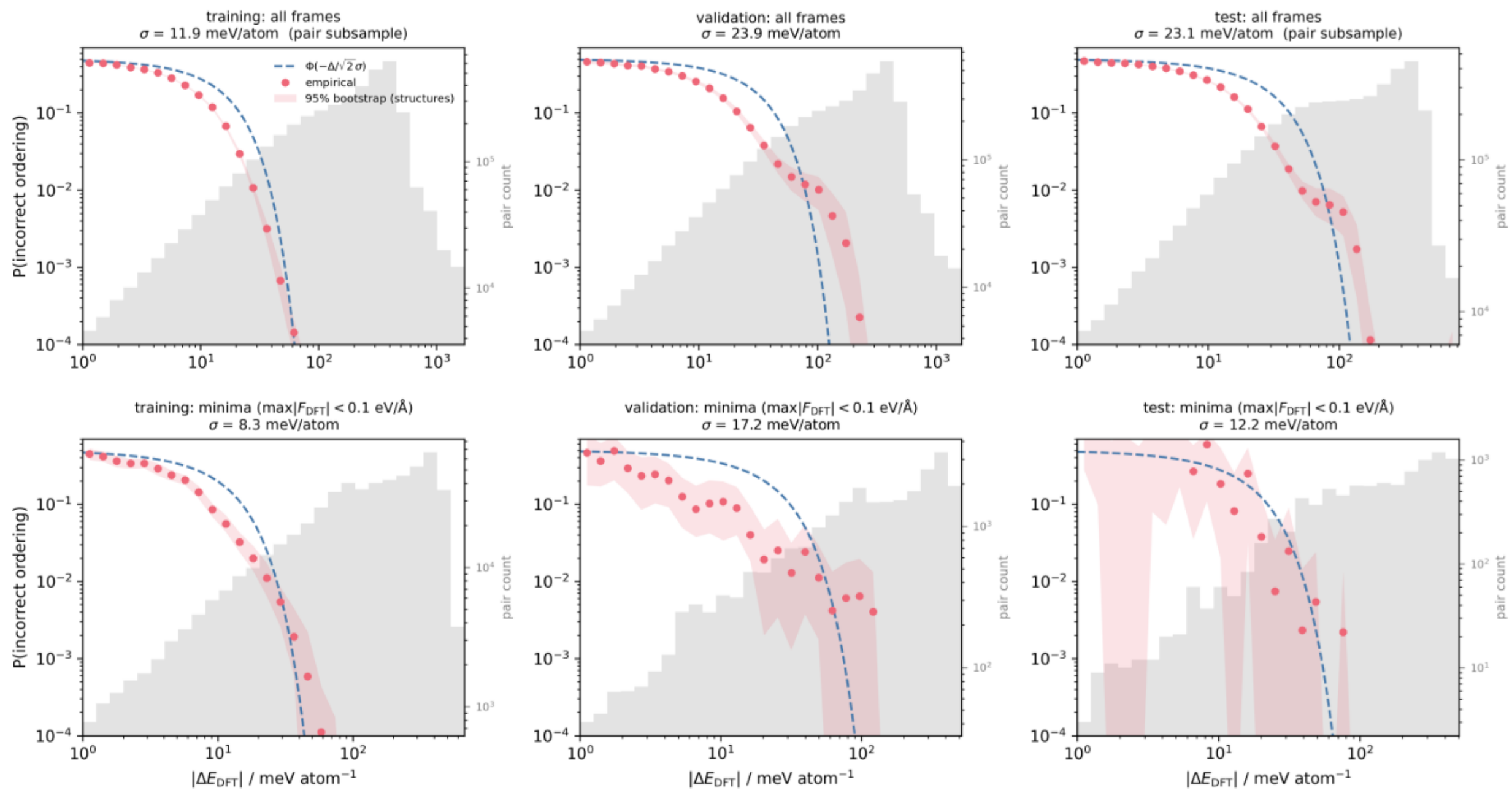

Figure S6. The probability of incorrect energy ordering of a given pair of structures by the MLIP is shown as a function of their true energy separation. The red circles show the empirical probability of misordering, computed for log-spaced bins. The red-shaded band shows a 95% confidence interval estimated via bootstrapping using 200 repeats, with each repeat using 139 randomly drawn structures. The blue dashed curve represents the prediction based on a simple error model, which assumes that DFT and ML energies are related by $E_{ML,i} = E_{DFT,i} + \varepsilon_i$ with $\varepsilon_i \sim N(0, \sigma^2)$. Under this model a pair with true gap Δ has $\Delta E_{ML} \sim N(\Delta, 2\sigma^2)$, so the misordering probability is **P(incorrect |Δ) = $\Phi(-\Delta/\sqrt{2}\sigma)$**, where Φ is the standard normal CDF. The empirical curves track this prediction reasonably closely, lying slightly below it for Δ <50 meV/atom range, and decaying more slowly at large gaps. The most likely reason for this is thay the errors of structurally similar (hence energetically close) configurations are correlated so the predicted relative energy benefits from error cancellation. Pairs separated by more than ≈ 9–27 meV/atom (population dependent; ≈ 15–45 kJ/mol per formula unit) are ordered correctly ≥ 90 % of the time.

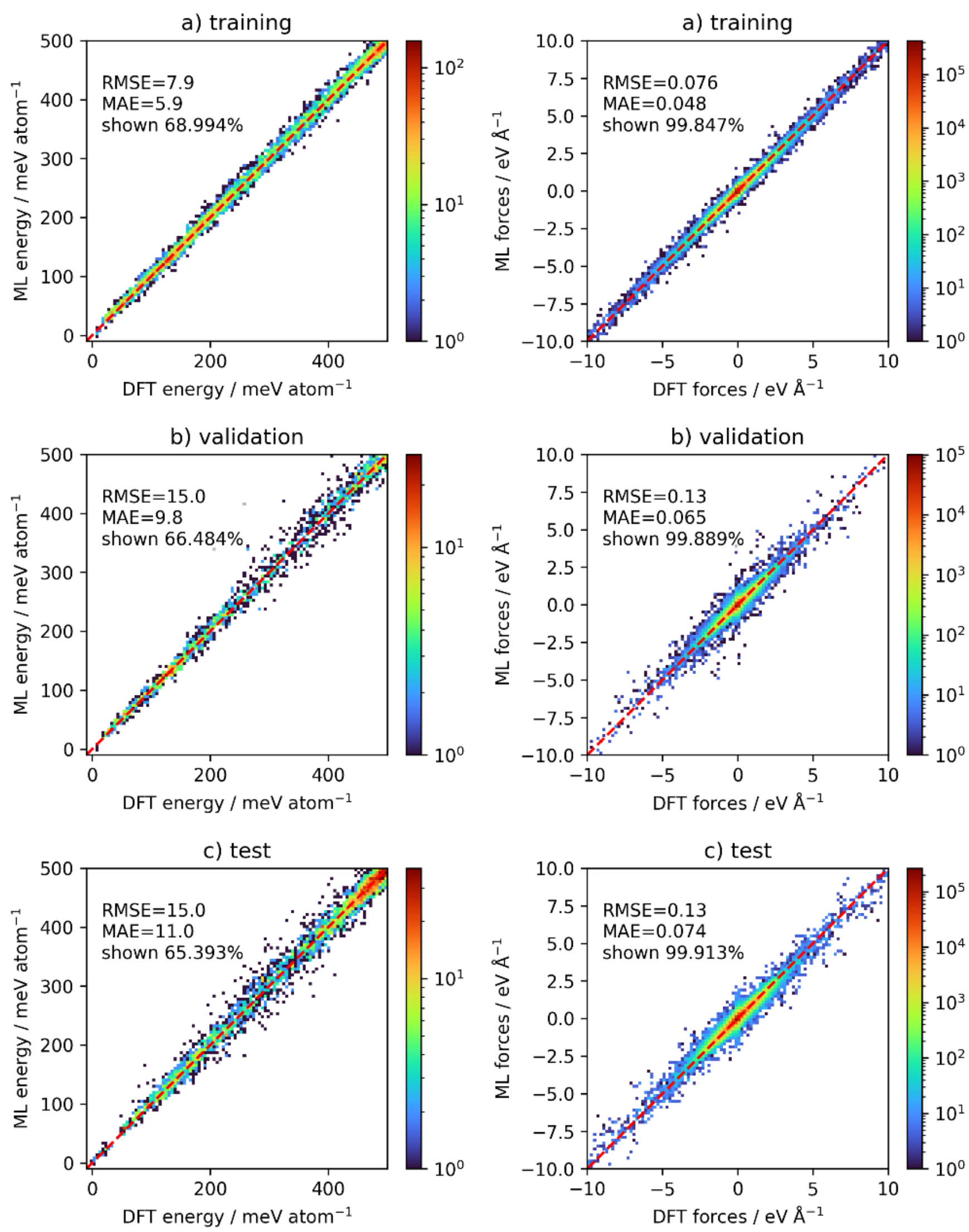

Figure S7. The magnified version of the ML vs DFT parity plots from the manuscript Figure 2, focusing on the energies up to 500 meV/atom and forces within ± 10 eV/Å. The colored vertical bar represents the abundance of energy and force values within the given dataset. Statistical values are given only for the datapoints within the plot boundaries, hence the difference from the complete dataset shown in Figure 2. RMSE = root mean square error, MAE = mean absolute error

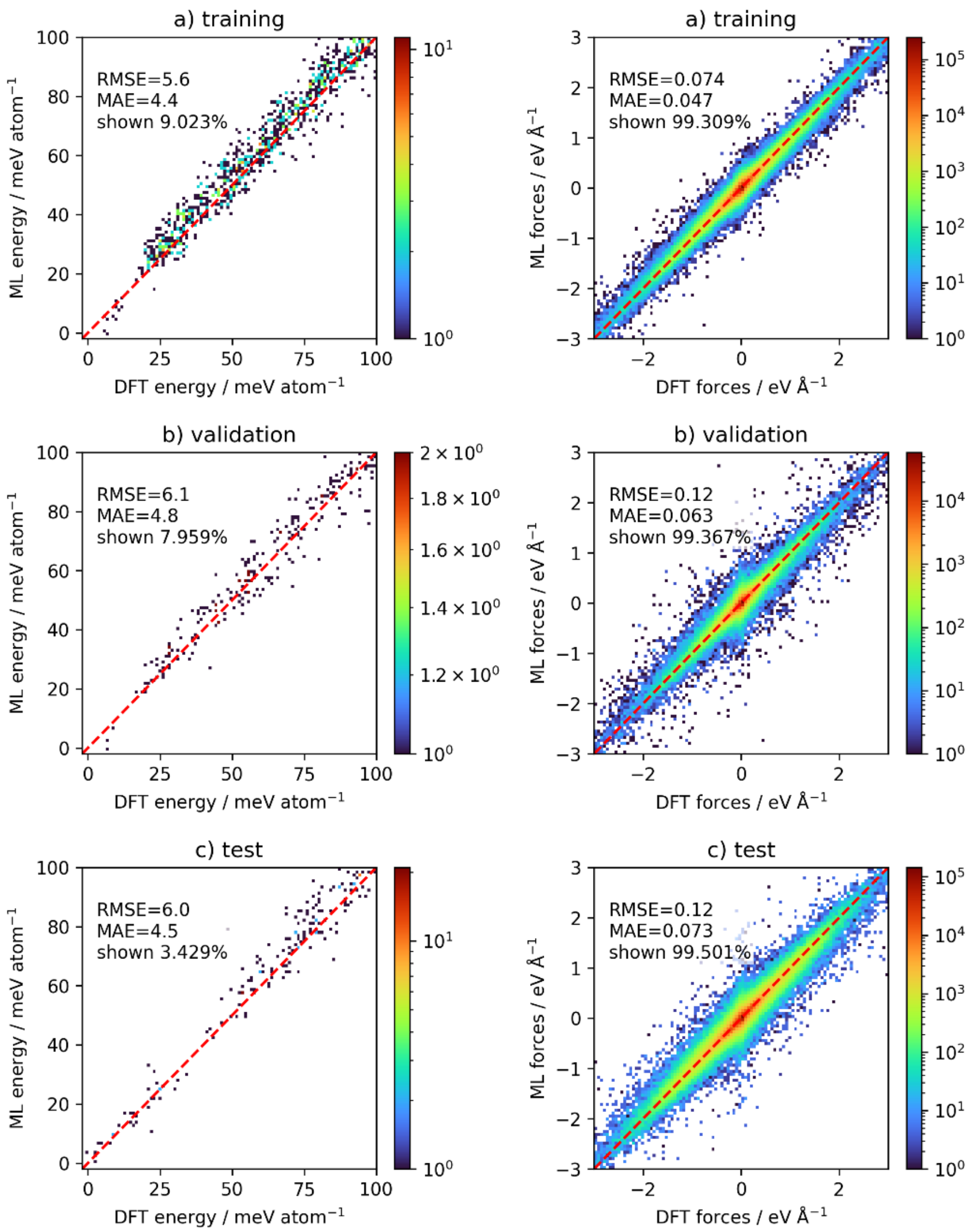

Figure S8. The magnified version of the ML vs DFT parity plots from the manuscript Figure 2, focusing on the energies up to 500 meV/atom and forces within ± 10 eV/Å. The colored vertical bar represents the abundance of energy and force values within the given dataset. Statistical values are given only for the datapoints within the plot boundaries, hence the difference from the complete dataset shown in Figure 2. RMSE = root mean square error, MAE = mean absolute error

S1.4 Geometry optimization and energy ranking using machine learned potentials

The forces and energy MLIPs were used to perform geometry optimization and energy ranking of ZIF structures containing 6 to 16 formula units per primitive cell, which would be too computationally expensive to study by periodic DFT. All putative hundreds and thousands ZIF structures were generated by the aforementioned AIRSS+WAM method. Subsequently, each structure was optimized with the MLIP trained on atomic forces via ASE's geometry optimization function within the FrechetCellFilter class. Symmetry was constrained to a tolerance of 0.01 Å. The force tolerance for the initial geometry optimization was set to 0.05 eV $Å^{-1}$. Then, the energy MLIP was applied for single point calculations for each optimized structure. All optimized structures were ranked by ascending energies, where the ones fall into an energy window of 45 kJ mol^{-1} with respect to the global minimum structure were selected for further analysis. Furthermore, ZIF structures containing 1 to 4 formula units per primitive cell, which were used to train the two MLIPs, were also processed from the same procedure. In a CSP search, the presence of duplicate structures provides a major indication for the convergence of dataset, where sufficient amount of structures has been searched.

The software of Critic2[9,10] with the built-in function "Compare reduce 3e-2", in order to compare thousands of ML-optimized structures. The "reduce" option allow the algorithm to omit structures already shown to be equivalent to the others in the list. The tolerance for the comparison method to identify two duplicate structures was set to 3e-2. Consequently, a full set of ML-optimized unique structures containing 1 to 16 formula units per primitive were obtained. Followed by clustering, the final set of structures were re-optimized with a tighter force tolerance of 0.005 eV $Å^{-1}$, and the single-point energy calculations were subsequently performed for each structure.

Since the cutoff distance for pairwise interactions was set to 5 Å, it was shortly realized that some 1D and 2D structures inherited channels and layers separation distances significantly larger (i.e. 10 Å) than is chemically plausible. Therefore, 646 1D and 2D ML-optimized structures that exhibit minimal pore radius of 5 Å or above, were selected for additional calculations with external stress. These structures were optimized first with the force MLIP, under the stress of 1 GPa and force tolerance of 0.005 eV $Å^{-1}$. This resulted in the reduction of interlayer/interchain spacing, within the MLIP interaction cutoff. The structures were then reoptimized under zero pressure, to make them consistent with the remaining structures that did not require special treatment. Finally, the energy MLIP was used to obtain the single-point energy of each structure. Clustering using the simulated PXRD comparison, implemented in Critic2, was performed to remove any newly formed duplicate structures.

S1.5 Post-processing of the predicted crystal structures

The final set of 9626 ML-optimized ZIF structures were obtained and ready for further analysis. The software PLATON[11] was used to convert all structures into the conventional crystallographic setting via the command ADDSYM EXACT SHELX. The structural void volume and packing coefficient of each structure were extracted from the command CALC VOID in PLATON.

All pore-related properties such as total surface area per mass, network accessible surface area per mass, total helium volume, pore limiting diameter, max pore diameter and number of percolated dimensions were evaluated through the Pore Analyzer function from the CCDC packages. The CSD Python API[12] was employed for high throughput analysis for all CSP predicted structures.

The software ToposPro[13] was used to determine the network topologies and dimension for all the structures in the final CSP set, via the default settings of the AutoCN module. Subsequently, the resulting nets were simplified and analyzed with the default settings of the ADS module. Finally, the obtained topological descriptors were searched through the built-in TTD database, as well as http://topcryst.com/ online server.

CSD Editor software was used to construct a database out of the predicted structures, that was then analyzed in ConQuest[14] to determine bond length and bond angle distribution statistics.

S1.6 Protocol for comparing experimental PXRD patterns with predicted CSP structures via Critic2

As a prerequisite for the comparison protocol, the experimental PXRD patterns were converted to .xy format, and all CSP predicted structures were supplied in .res file format. The open source software PowDLL was used to convert experimental PXRD patterns from .raw, .xrdml or .brml files to .xy format. Subsequent analysis involved several steps in Critic2:

1) The background of the experimental PXRD pattern was calculated via the command "XRPD BACKGROUND experimental_pattern.xy background.xy".
2) The command "XRPD FIT background.xy" was used to obtain a list of background subtracted reflections and intensities.
3) The command COMPAREVC was used to compare the experimental PXRD pattern with each CSP predicted structure.

Overall, 9626 individual comparisons between all ML-optimized structures and the experimental PXRD pattern were performed, on the PLGrid high performance computer (HPC) HPC Helios. Finally, all CSP predicted structures were ranked by ascending DIFF scores, of which the structures with lower DIFF scores will be investigated further for possible experimental matching structures.

S1.7 Periodic DFT optimization of the structures deemed likely experimental candidates

The 982 predicted structures deemed to be synthesizable based on the energy-porosity criterion (Section S7) were geometry-optimized with periodic DFT. These calculations used PBE functional, combined with Grimme D3 dispersion correction.[15] The plane-wave basis set truncated at 800 eV cutoff, and CASTEP default ultrasoft pseudopotentials were used. The electronic k-point grid was sampled with a $2\pi x 0.06$ Å^{-1} Monkhorst-Pack k-point grid.[4] Convergence criteria were set as follows: maximum energy change 2×10^{-5} eV atom^{-1}; maximum atomic force 0.05 eV Å^{-1}; maximum atom displacement 10^{-3} Å; maximum residual stress 0.05 GPa.

S2. The use of Separate MLIPs for Energies and Forces

In this work two distinct MLIP's were trained to reproduce the DFT energies and forces respectively, rather than training a single model to reproduce both, as is common practice. This decision was made as in initial testing models targeting both energies and forces achieved very poor performance with approximately 10 times larger MAEs compared to separate models. After spending significant effort attempting to resolve this issue we proceeded using separate models. This decision was made pragmatically, as the MLIPs were used purely as a tool to accelerate the CSP, and the models trained separately to energies and forces where accurate enough to usefully rank the structures - MAEs of ~12 meV/atom and 66 meV $Å^{-1}$. For clarity, the MLIP trained on forces was used for structural relaxations whilst the MLIP trained on energies was then used for final energy rankings.

Subsequently this issue was revisited and, after updating the supplied neighbor list, a single MLIP was successfully fit to energies and forces simultaneously. As such, we hypothesize that the previous advantage observed for separate models was due to the energy and force training data appearing inconsistent and stress that in general there is no need for separate models.

In addition to the above, as fitting to energies and forces with separate models is unusual, we also fit a MACE model[16] to both energies and forces to check that substantially better accuracy could not be achieved with a commonly used modern architecture and standard setup. The resulting parity plots for energy and force predictions on the training, validation and testing sets are shown in Figure S9. The achieved accuracy is very comparable to the separate MLIPs fit in this work. Furthermore, given the recent success of foundation models, we fit a second MACE model by fine-tuning the MACE-MPA-0 foundation model[17] using the recommended multi-head-replay strategy to prevent catastrophic forgetting. The fine-tuned model achieved extremely similar validation MAE's of 9 meV/atom and 87 meV $Å^{-1}$ (compared to 7 meV/atom and 84 meV $Å^{-1}$ with vanilla fitting), indicating that fine-tuning offers little benefit here. We suggest this is likely due to the relatively large amount of training data (10972 configurations containing 444,907 atoms), for a single composition.

In summary, neither MACE model offered a substantial improvement in accuracy compared to the separate MLIP's fit to energies and forces.

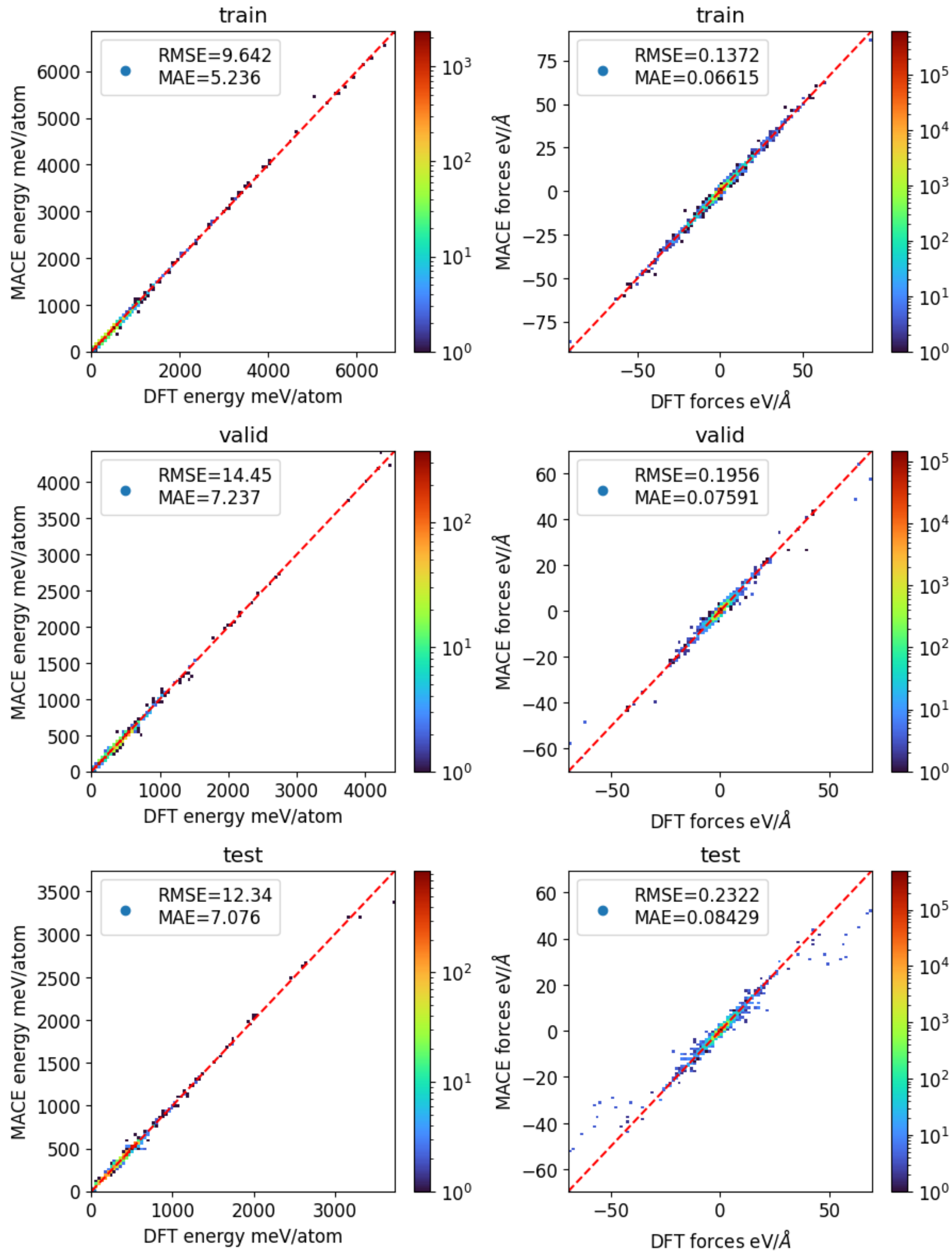

Figure S9. Parity plots of energies and forces comparing MACE predictions with reference data for the training, validation, and test sets. Errors are quantified using RMSE and MAE (shown inset). The energies were referenced to the lowest-energy structure, which was set to zero. RMSE = root mean square error, MAE = mean absolute error

S3. Mechanochemical synthesis

The experimental preparation of all materials used for the assignment of the PXRD data against the predicted structures was described in an earlier publication.[18] Ball milling reactions were conducted in a 14 mL jar with one 7 mm (1.4 g) and one 9 mm (3.5 g) diameter stainless steel ball bearing. In each liquid-assisted grinding (LAG) experiment, 100 µL of toluene, chloroform or dimethylformamide, depending on experiment (see Table S5), was added into a milling jar containing the ball bearings, zinc oxide (75.0 mg, 0.92 mmol) and imidazole (125.5 mg, 1.84 mmol). The samples were milled at 30 Hz for up to 90 minutes using a Retsch MM400 ball mill.

Guest-free porous material of unknown structure was prepared by the following procedure. Zinc oxide (75.0 mg, 0.922 mmol), imidazole (125.5 mg, 1.843 mmol) and 100 µL of acetophenone were placed in 14 mL Teflon jar along with one 7 mm (1.4 g) and one 9 mm (3.5 g) diameter stainless steel ball bearing. Reaction mixture was milled for 1 hour at a frequency of 30 Hz in an InSolido Technologies IST500 mixer mill. Product was washed with acetone and then dried for 3 hours at 150 °C in a vacuum oven.

S4. Rietveld refinement

Rietveld refinement[19] was performed in Topas Academic 7.[20] Predicted crystal structure Znimid2_16_56_Pccn_YMQOEPiX (#2470 in the Chemiscope file) was refined against the PXRD pattern recorded for the material obtained via heating of **crbA**-Zn**Im**$_2$ (CSD: GAKXOX) at 150 °C (see section S3). Diffraction peak shapes were described with a pseudo-Voigt function, background was modelled with a 6th degree Chebyshev polynomial function. Unit cell parameters and zero shift were refined, while atom positions were kept fixed at the coordinates obtained from the theoretical structure.

S5. Comparison of DFT and ML energies for the predicted structures

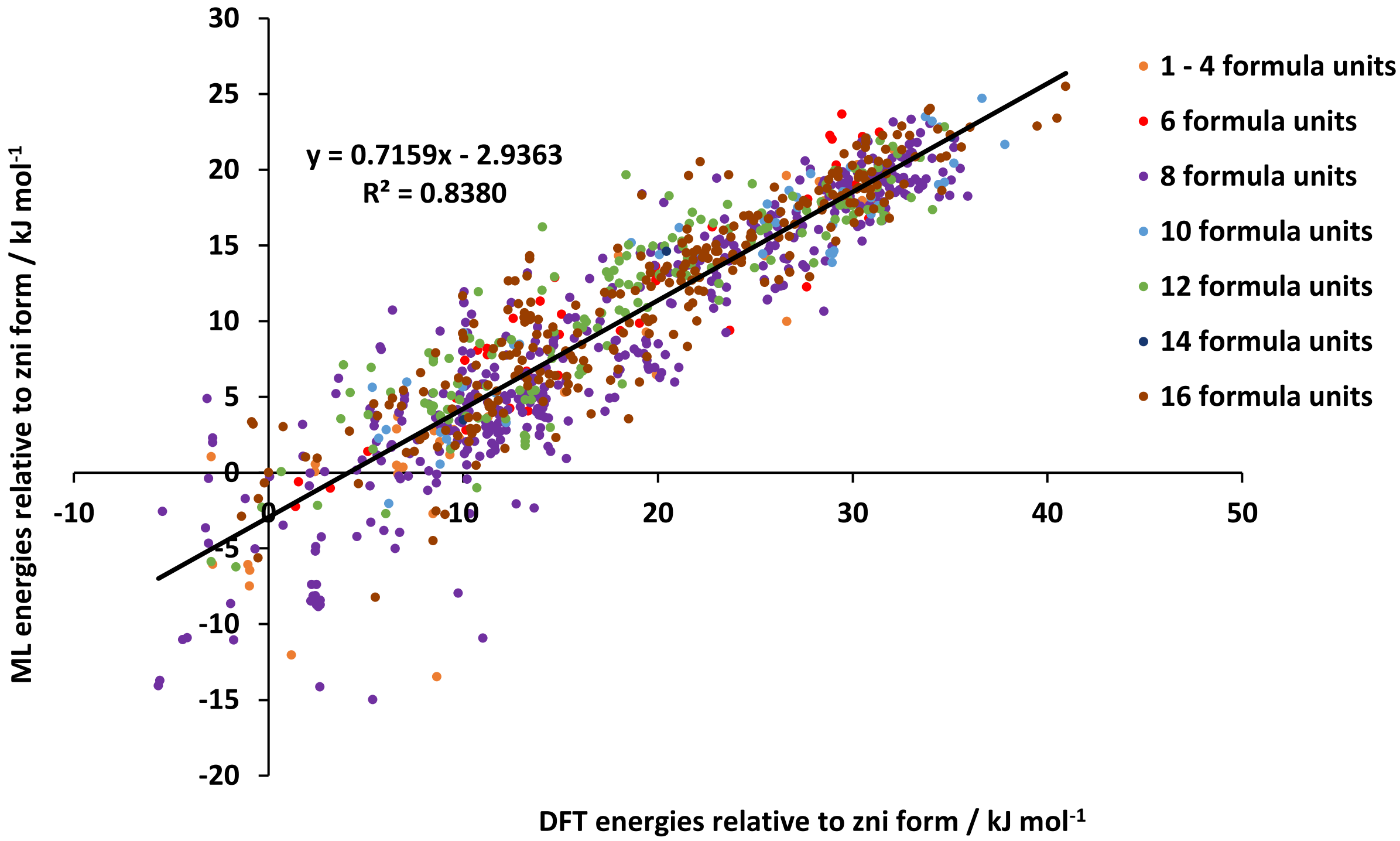

Figure S10. Plot of ML relative energies against the DFT relative energies. In both cases, the predicted structures matching with the experimental structure (CSD IMIDZB02) with **zni** topology was used as a reference (origin of the plot). The regression line is drawn based on the energies of all structures, while different colors represent show datapoints separated by the number of formula units.

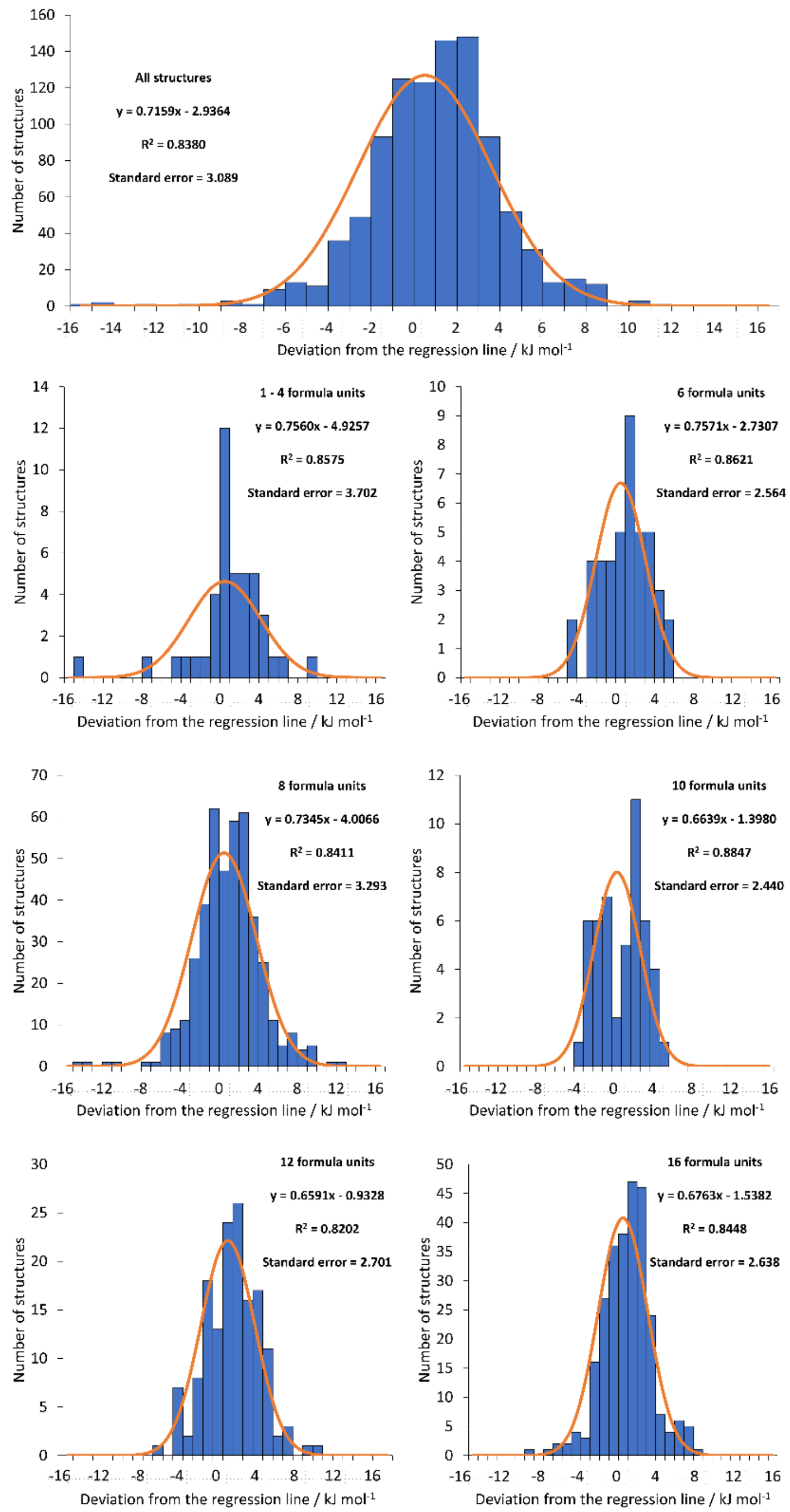

Figure S11. Deviations of relative ML energies from the regression line on Figure S11. At the top the overall distribution is shown, followed by the individual histograms grouped by the number of formula units. The orange curve in each histogram shows the normal distributions with the same standard deviation as the underlying data. It is evident that the pattern of energy deviations is similar across all groups, demonstrating that the MLIPs perform similarly for structures with all numbers of formula units per primitive cell.

S6. Geometry analysis and distribution of framework topologies within the energy landscape

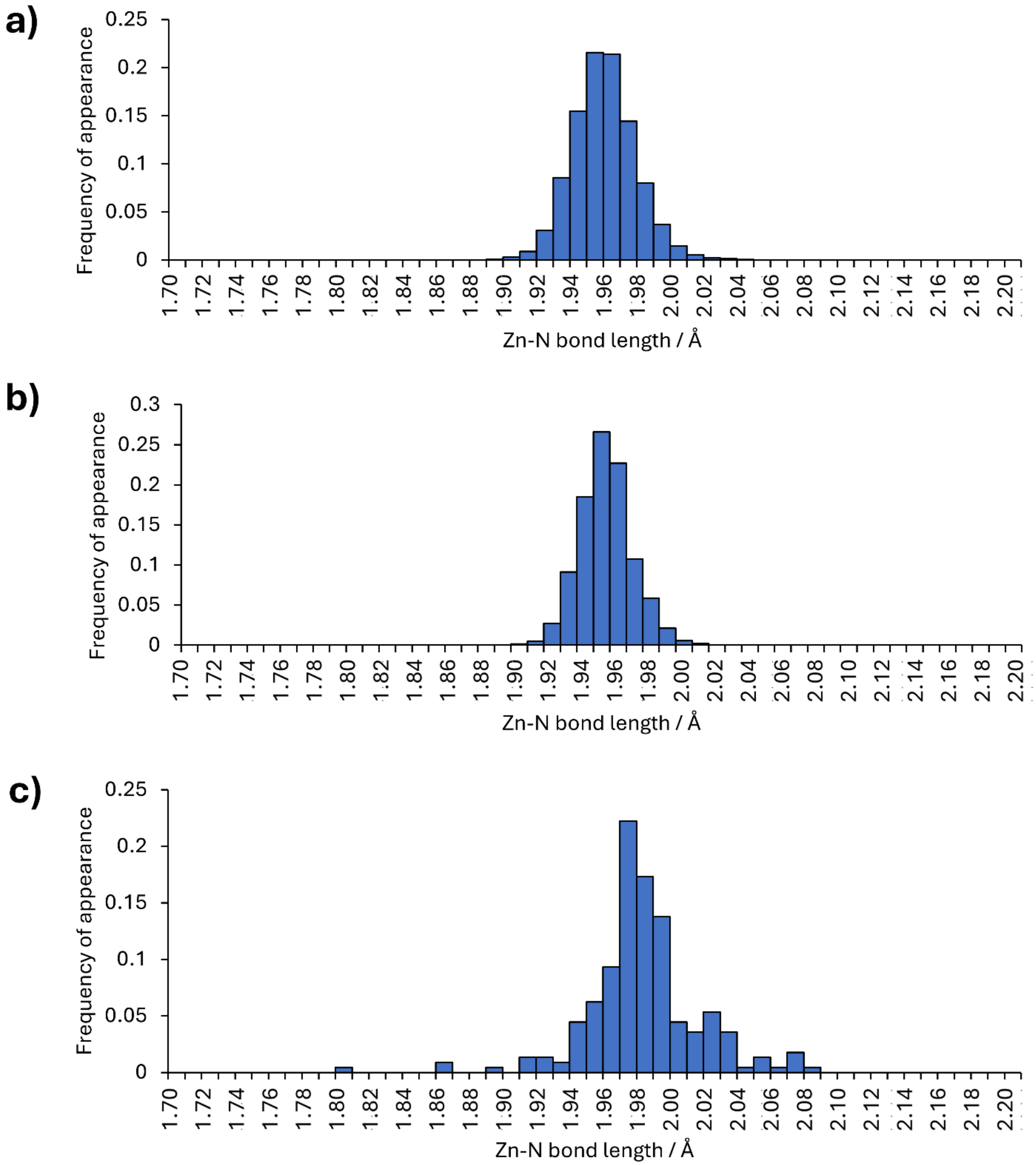

Figure S12. Comparison of the Zn-N bond length distributions: a) for all predicted structures; b) for the predicted structures from the likely-synthesizable group; c) for the experimental structures of Zn**Im**$_2$ found in CSD.

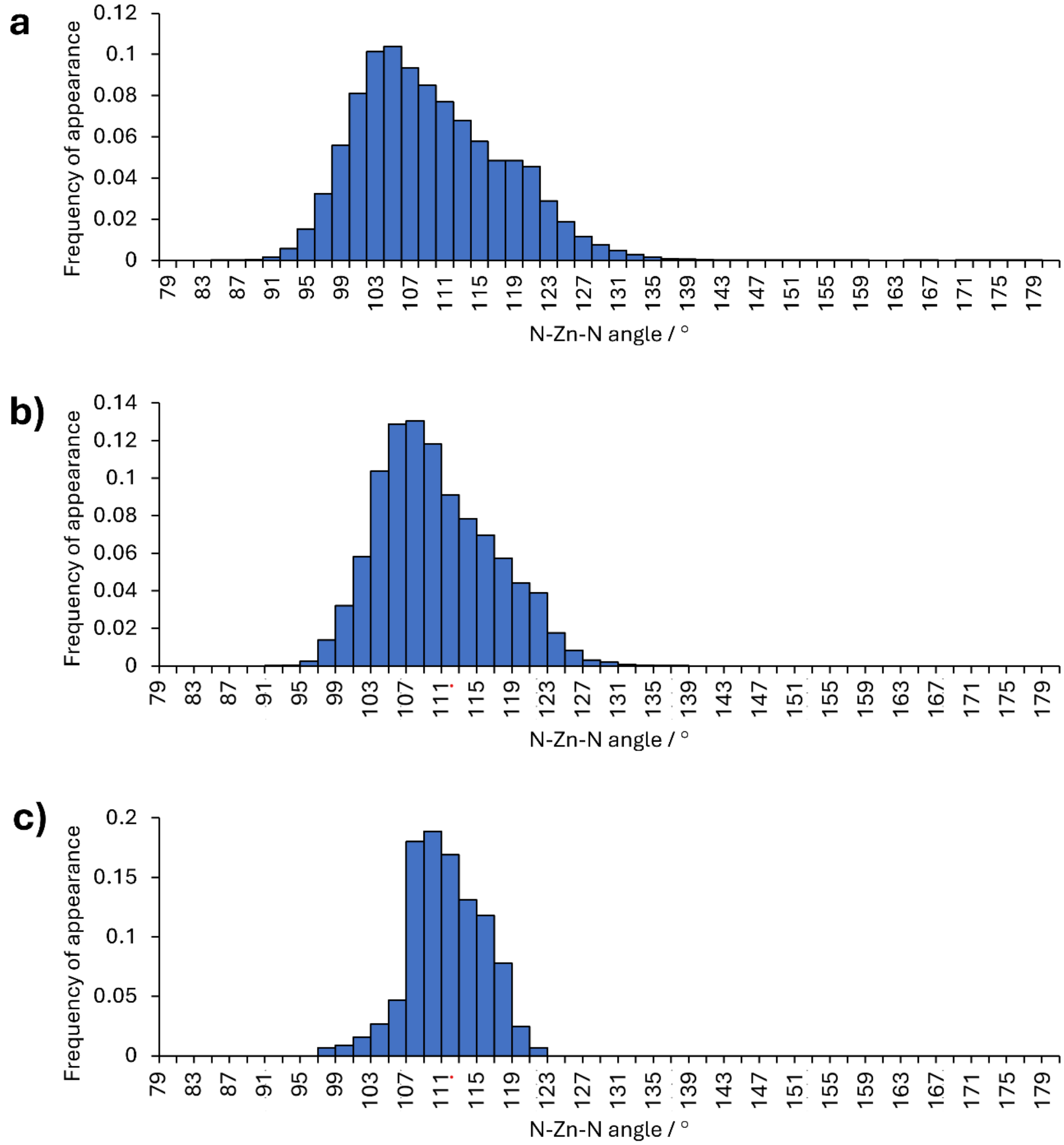

Figure S13. Comparison of the Zn-N bond length distributions: a) for all predicted structures; b) for the predicted structures from the likely-synthesizable group; c) for the experimental structures of Zn**Im**$_2$ found in CSD.

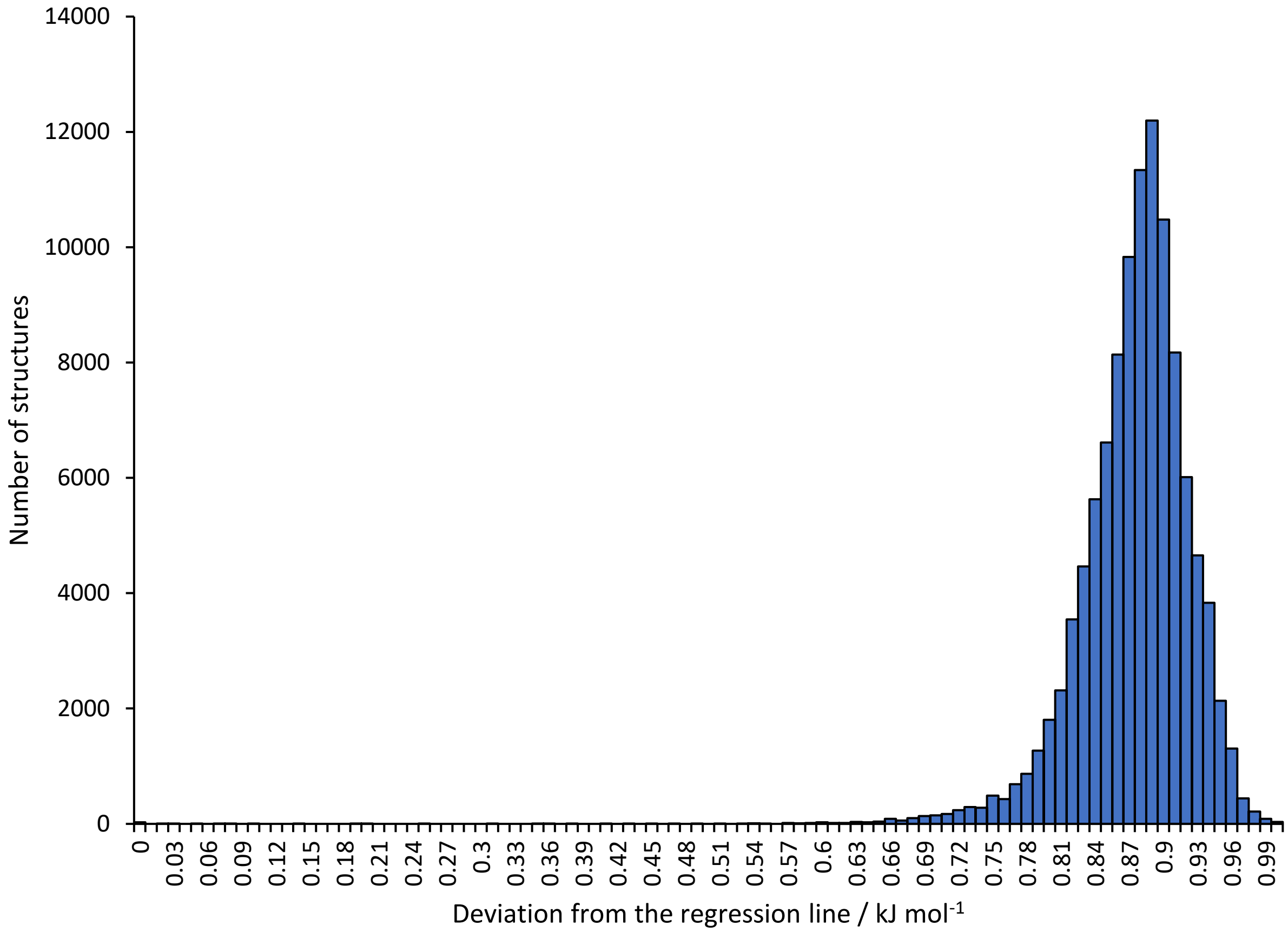

Figure S14. The τ geometry index calculated for all predicted structures. The characteristic values of τ are 0 for square planar geometry, 0.43 for seesaw geometry and 1 for tetrahedral geometry of the metal coordination center. The distribution maximum is located at 0.9, indicating that the coordination geometry in the predicted structures is predominantly tetrahedral, as would be expected for ZIF structures containing Zn nodes.

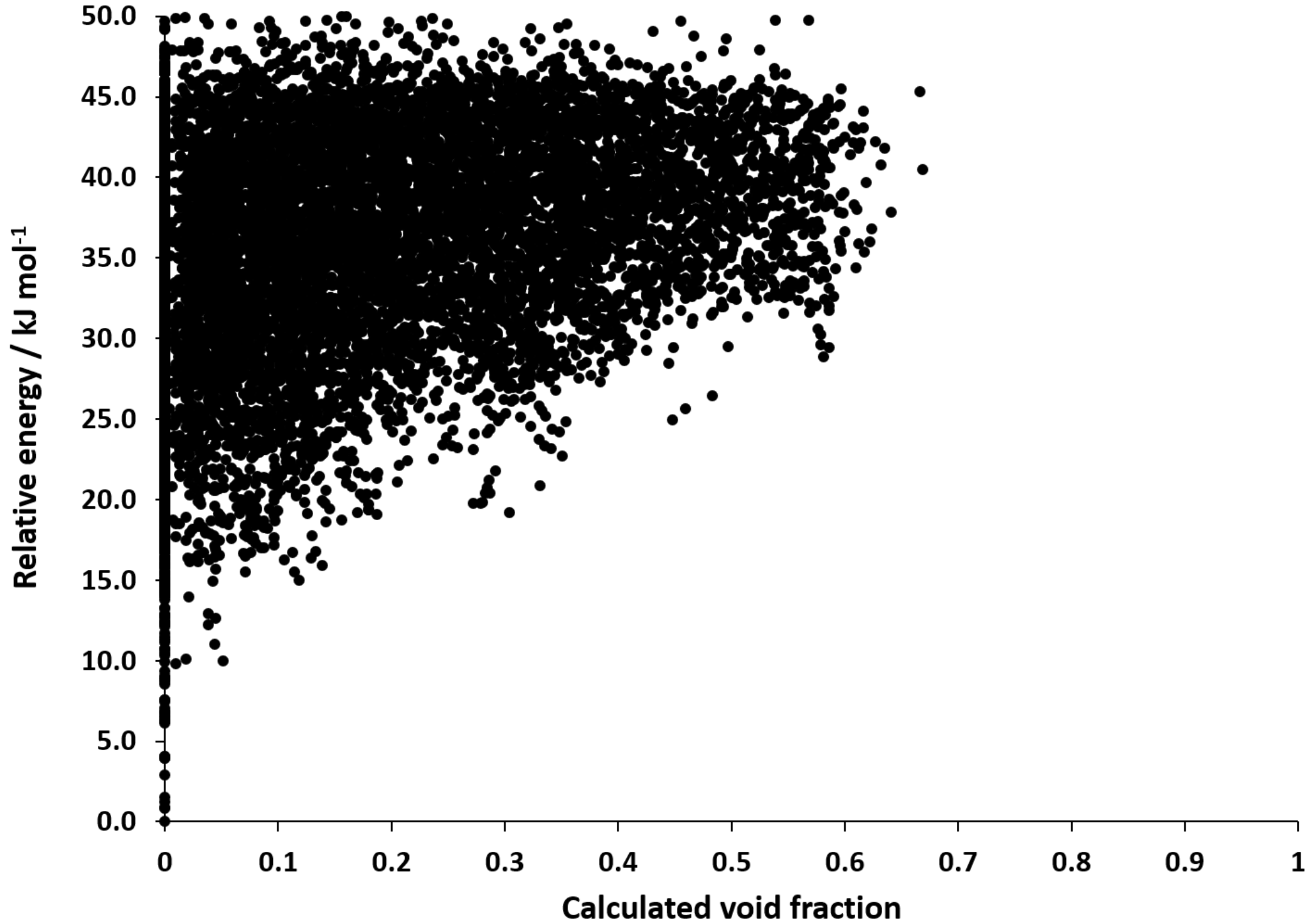

Figure S15. CSP energy landscape showing the relative energy of the predicted structures against the calculated void fraction.

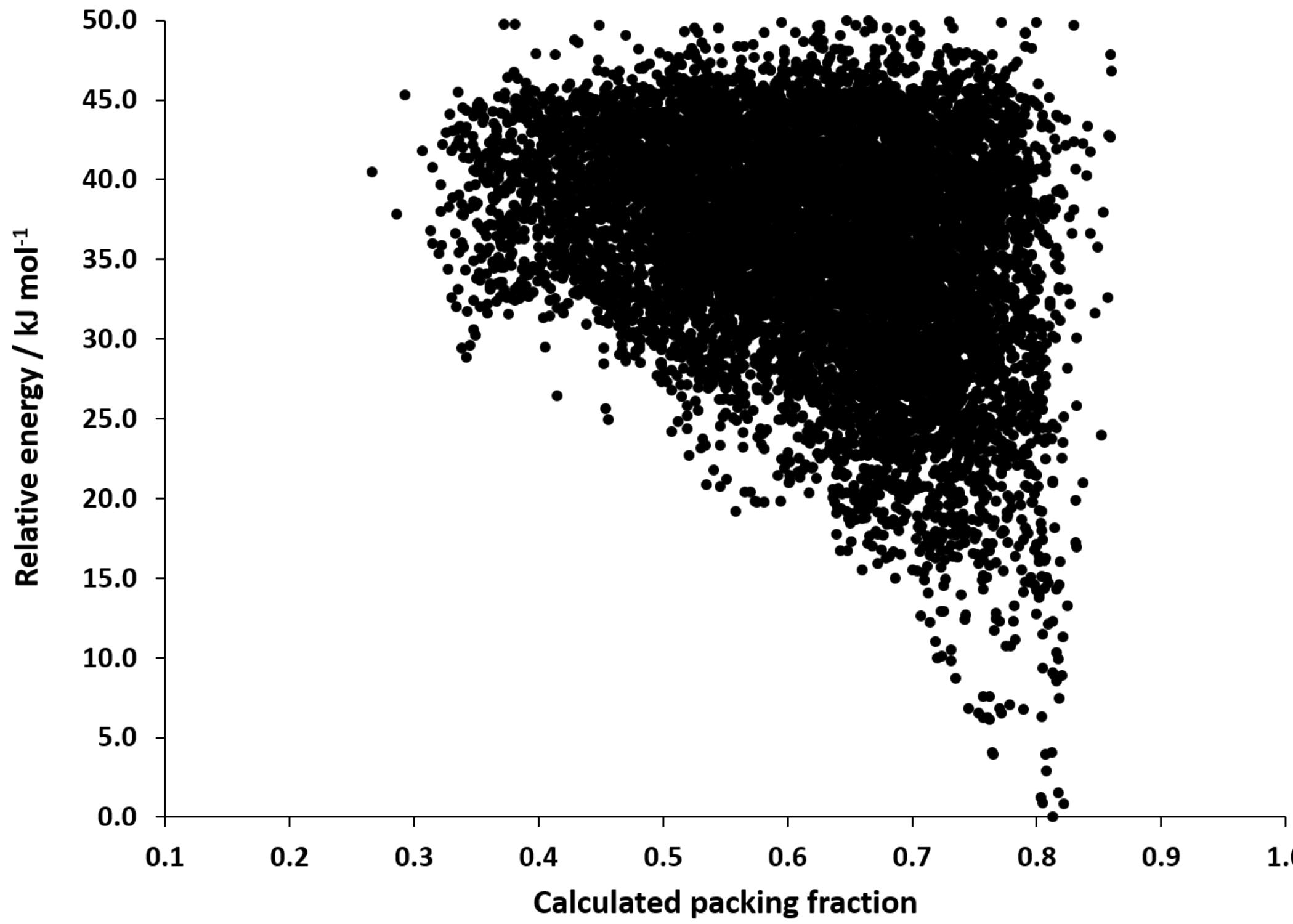

Figure S16. CSP energy landscape showing the relative energy of the predicted structures against the calculated packing fraction.

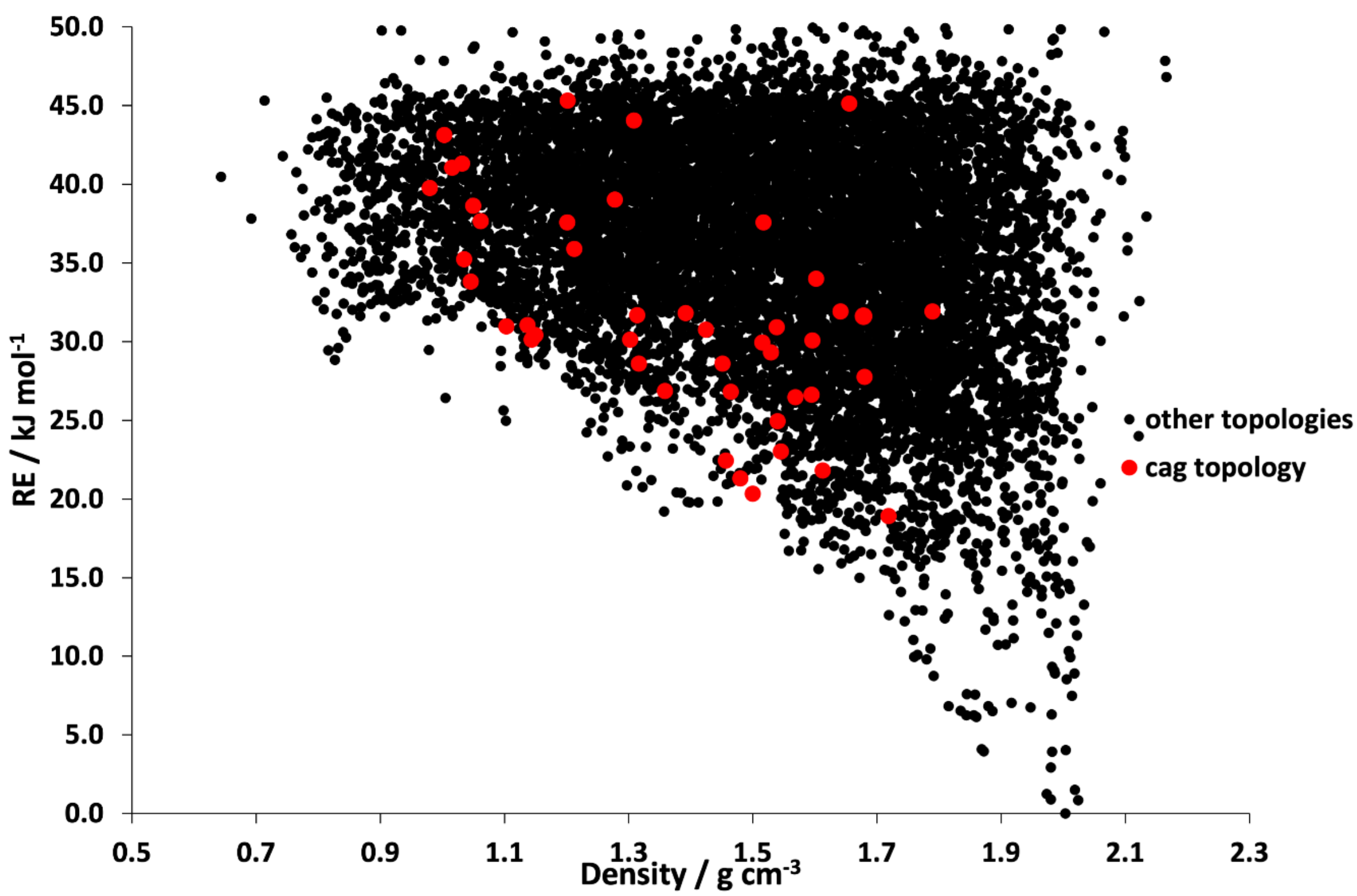

Figure S17. CSP energy landscape highlighting the structures with **cag** topology.

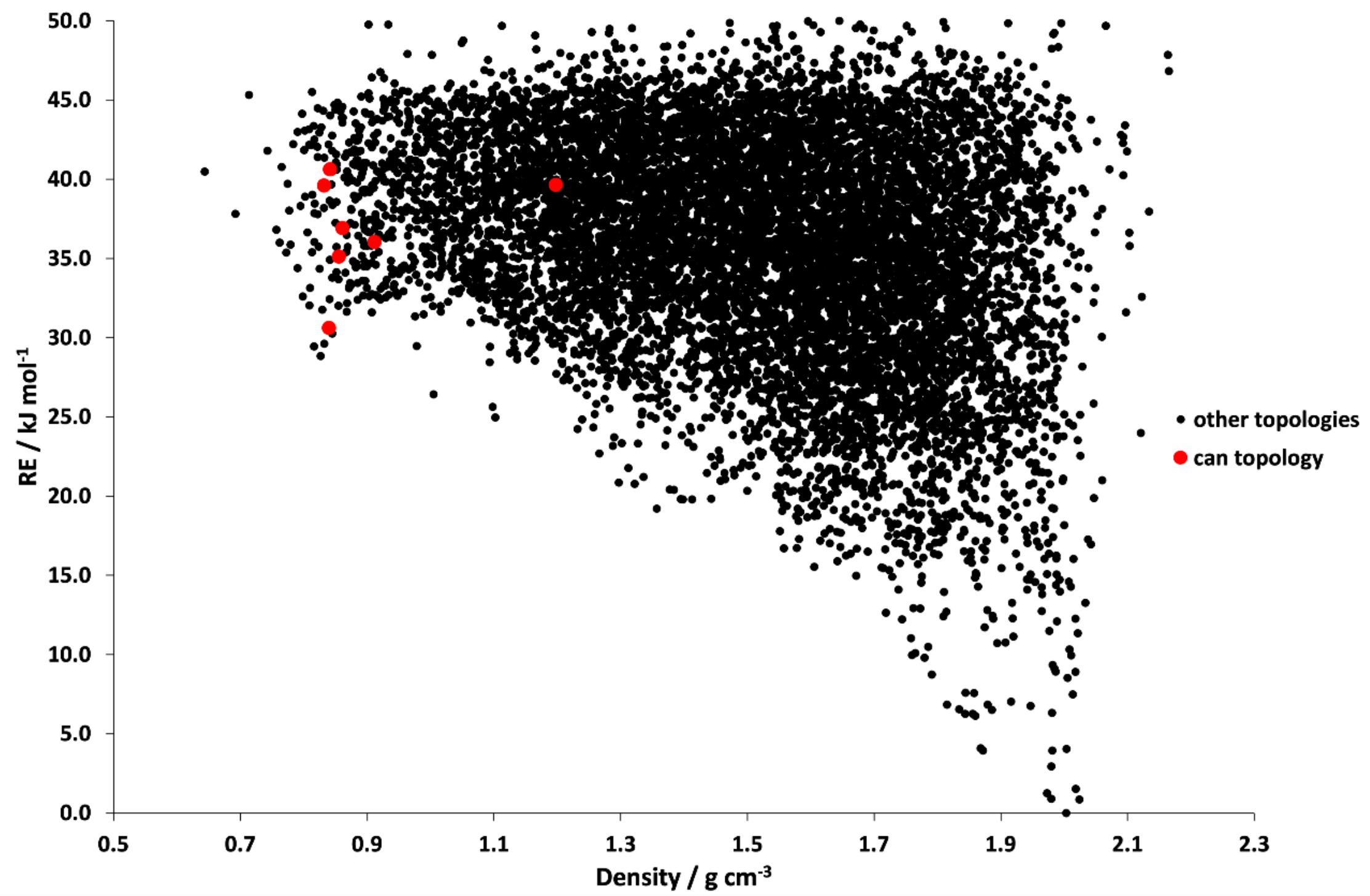

Figure S18. CSP energy landscape highlighting the structures with **can** topology.

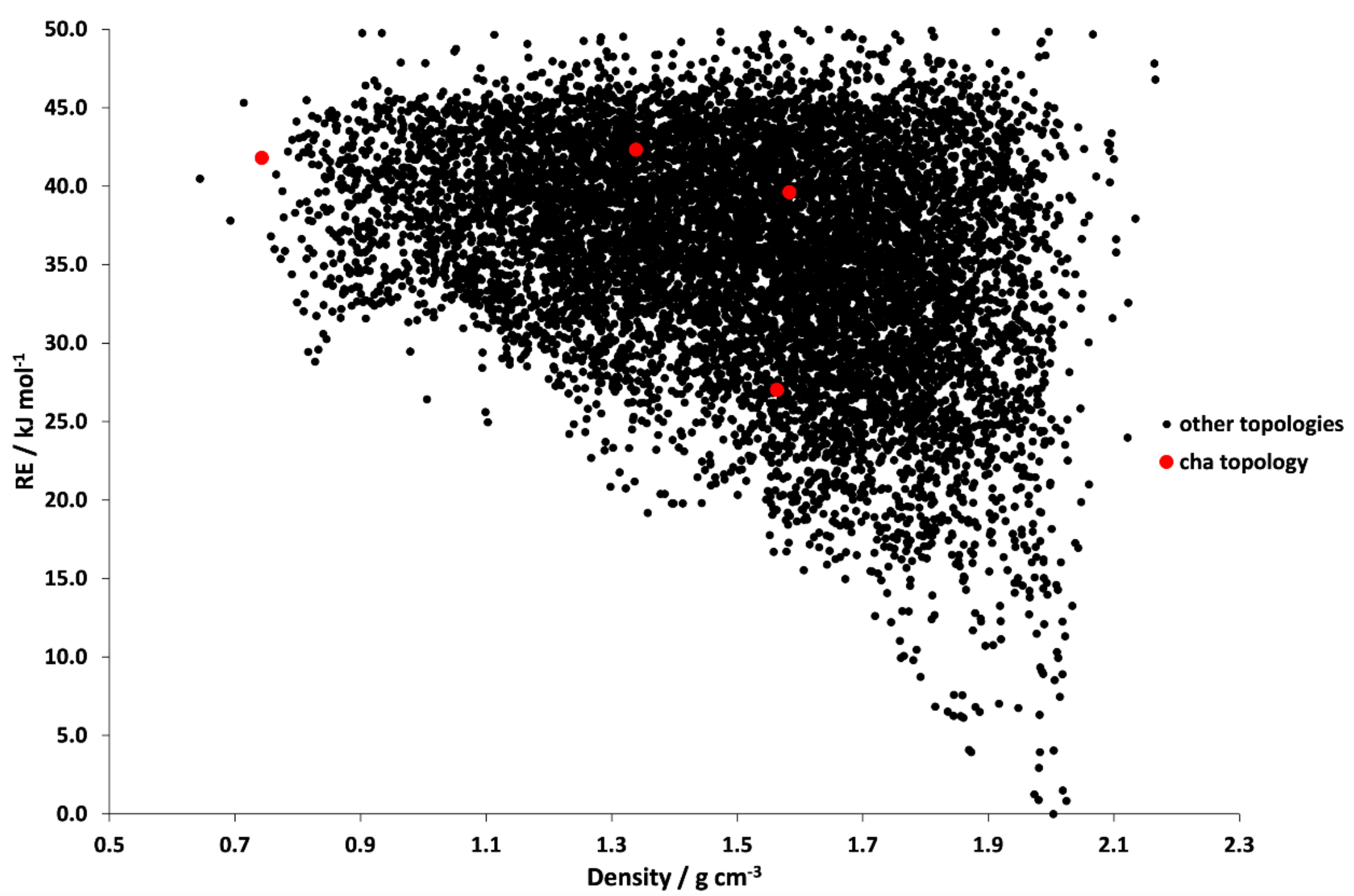

Figure S19. CSP energy landscape highlighting the structures with **cha** topology.

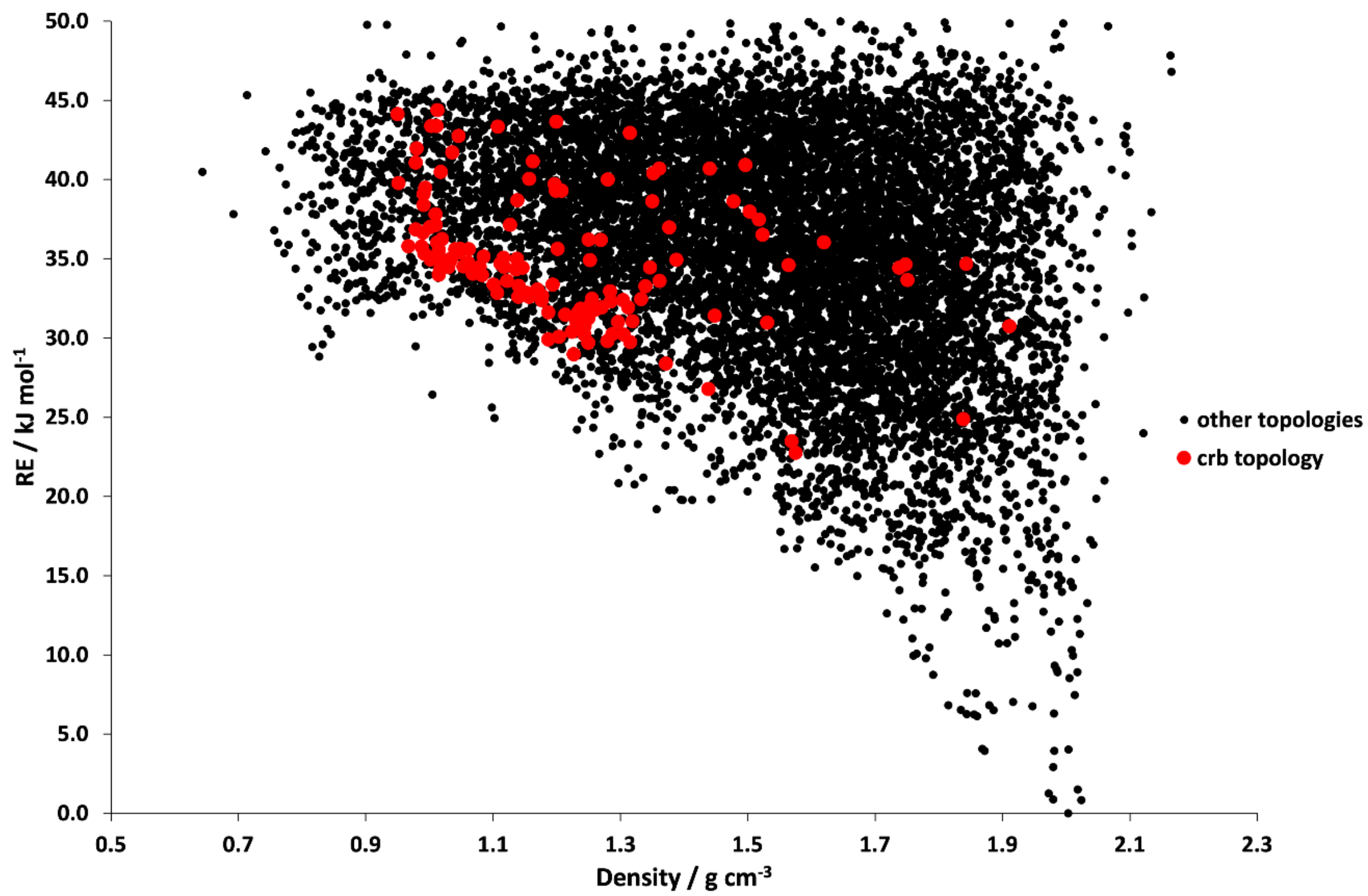

Figure S20. CSP energy landscape highlighting the structures with **crb** topology.

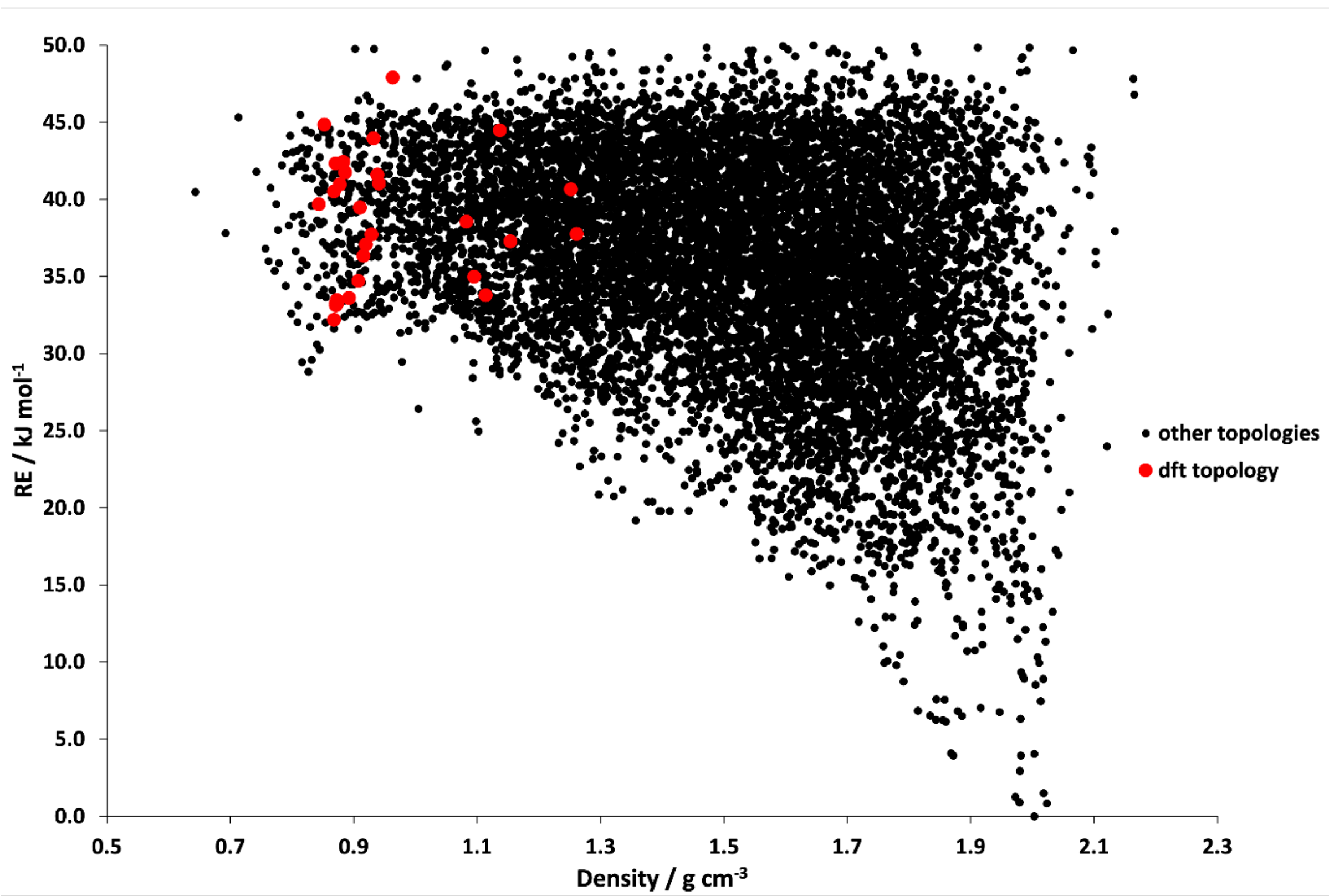

Figure S21. CSP energy landscape highlighting the structures with **dft** topology.

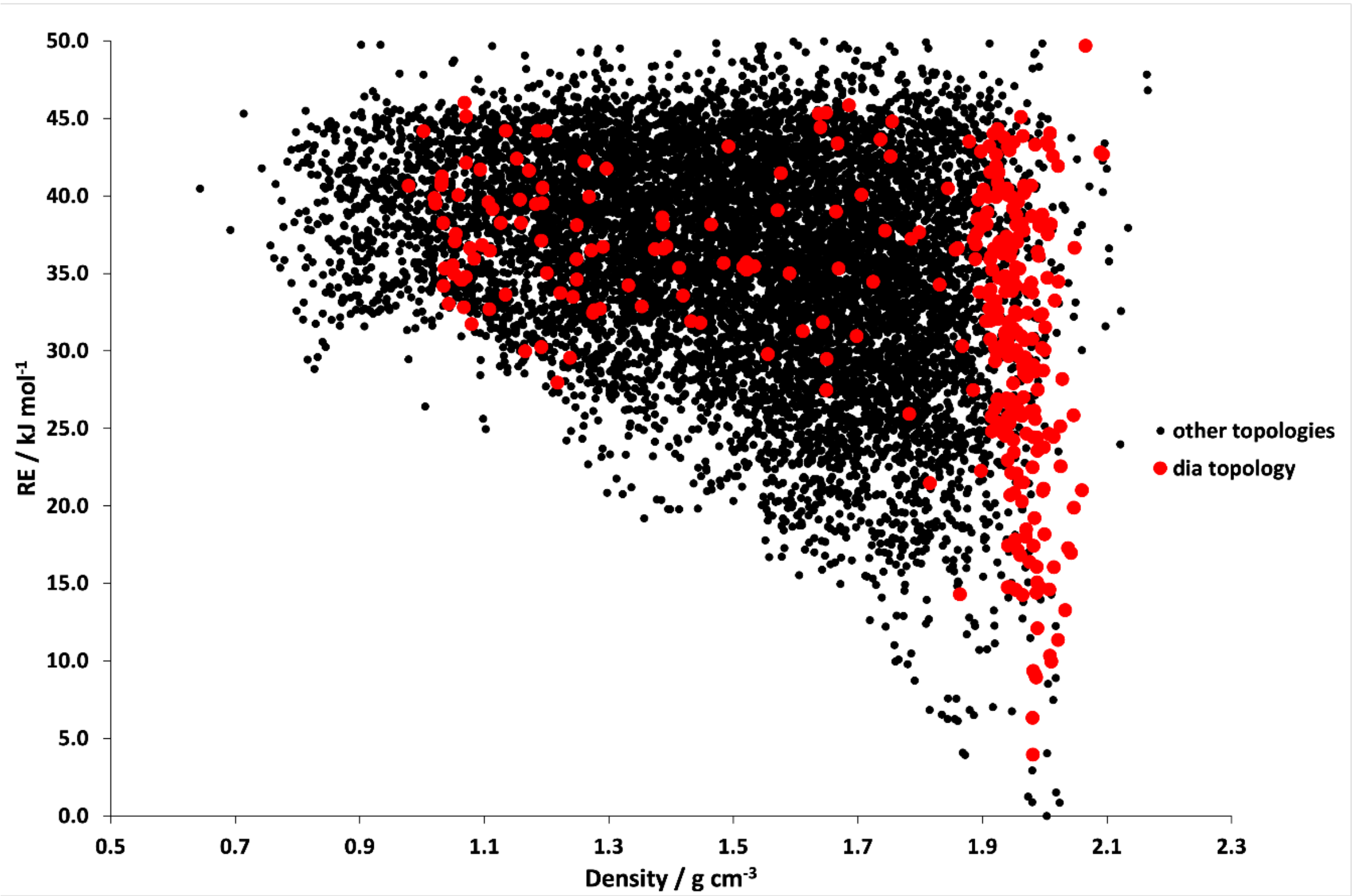

Figure S22. CSP energy landscape highlighting the structures with **dia** topology.

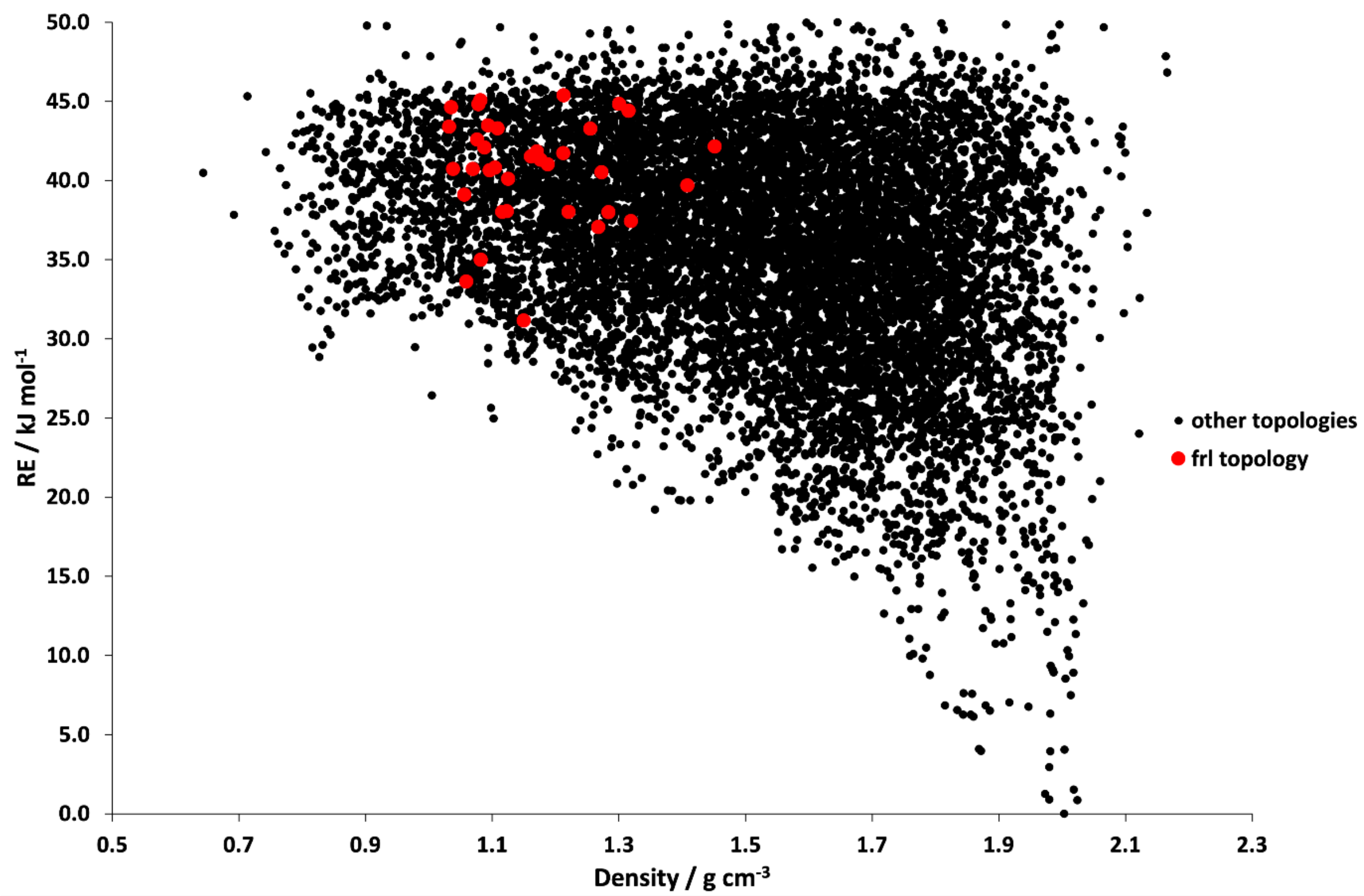

Figure S23. CSP energy landscape highlighting the structures with **frl** topology.

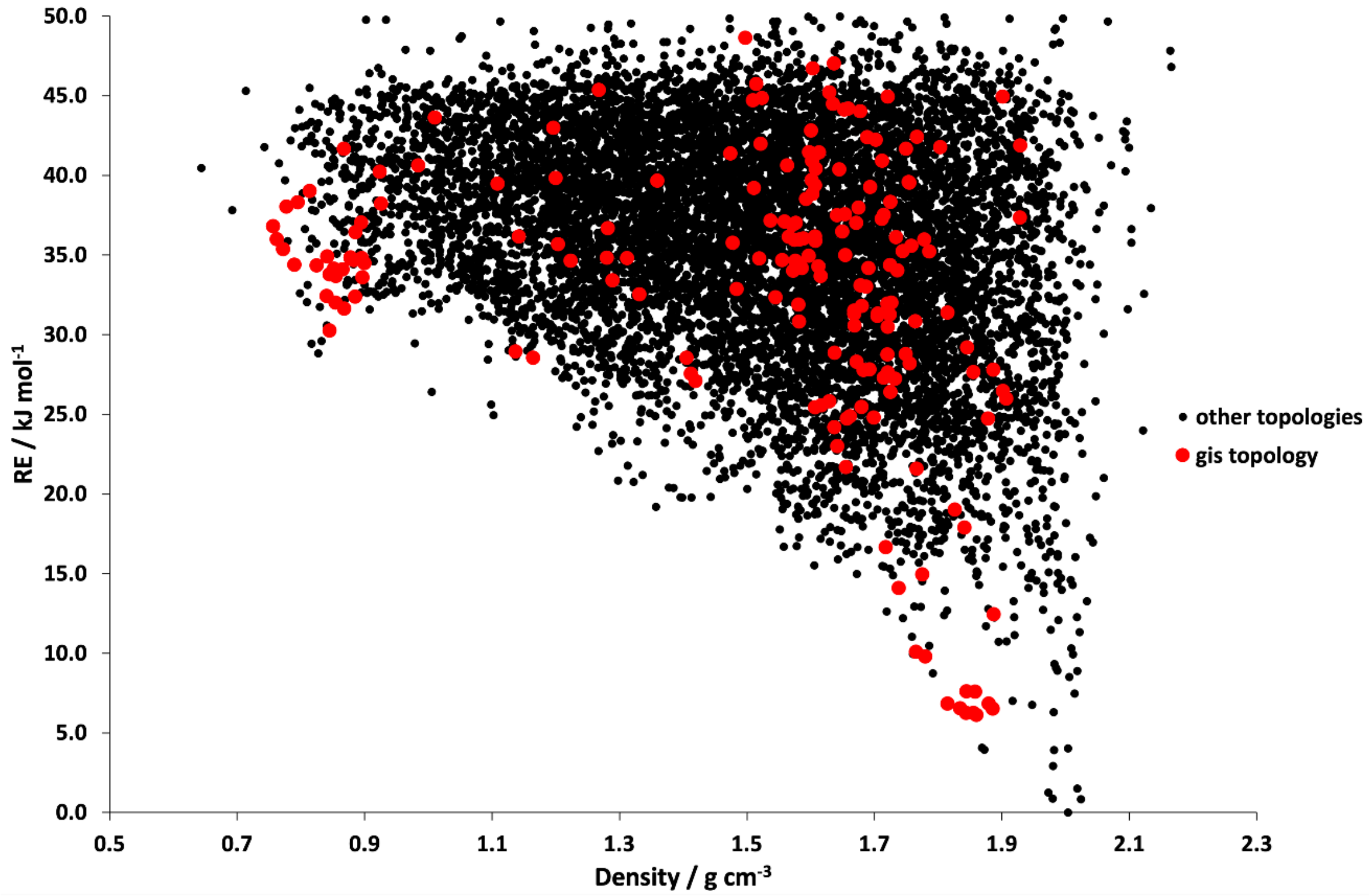

Figure S24. CSP energy landscape highlighting the structures with **gis** topology.

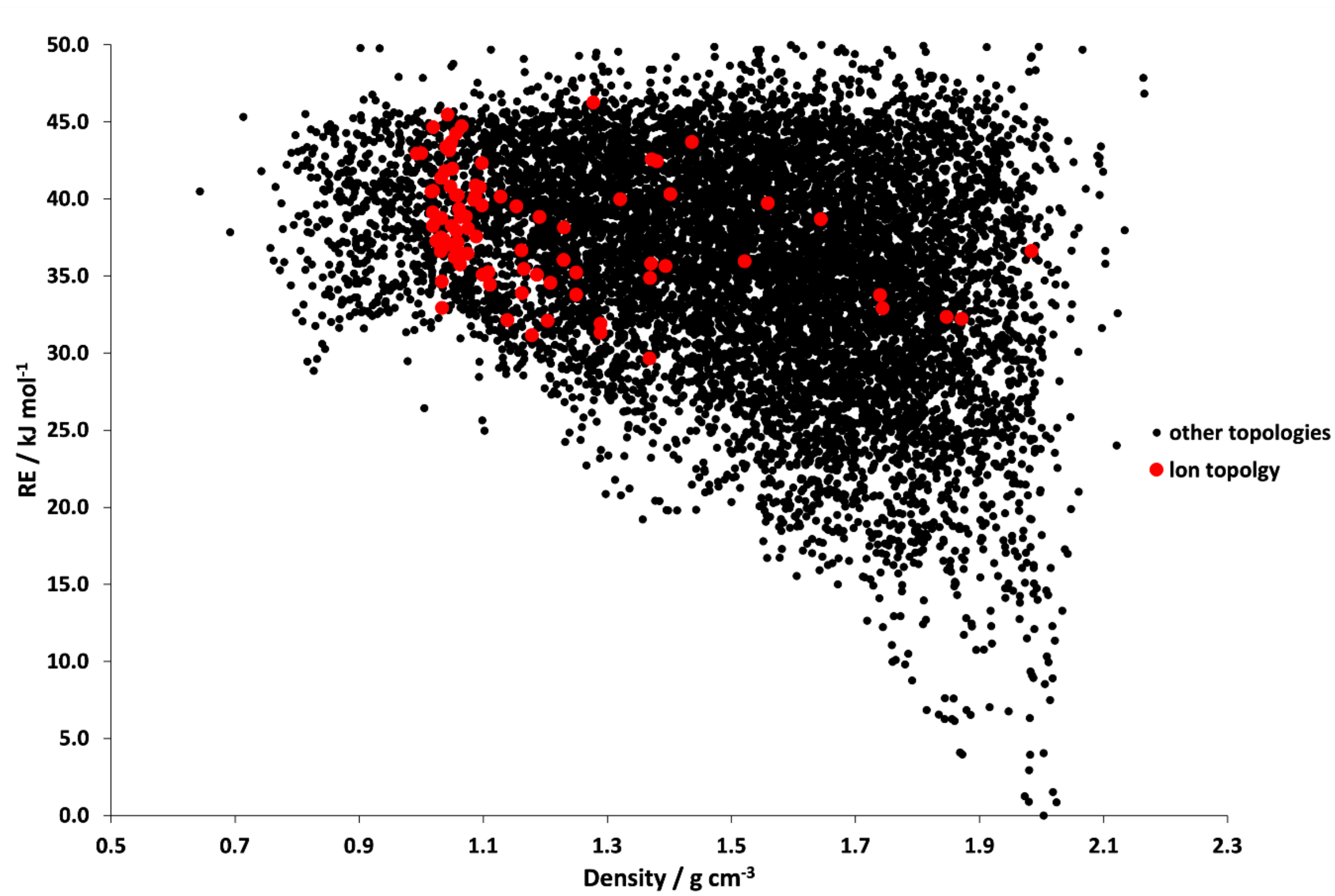

Figure S25. CSP energy landscape highlighting the structures with **lon** topology.

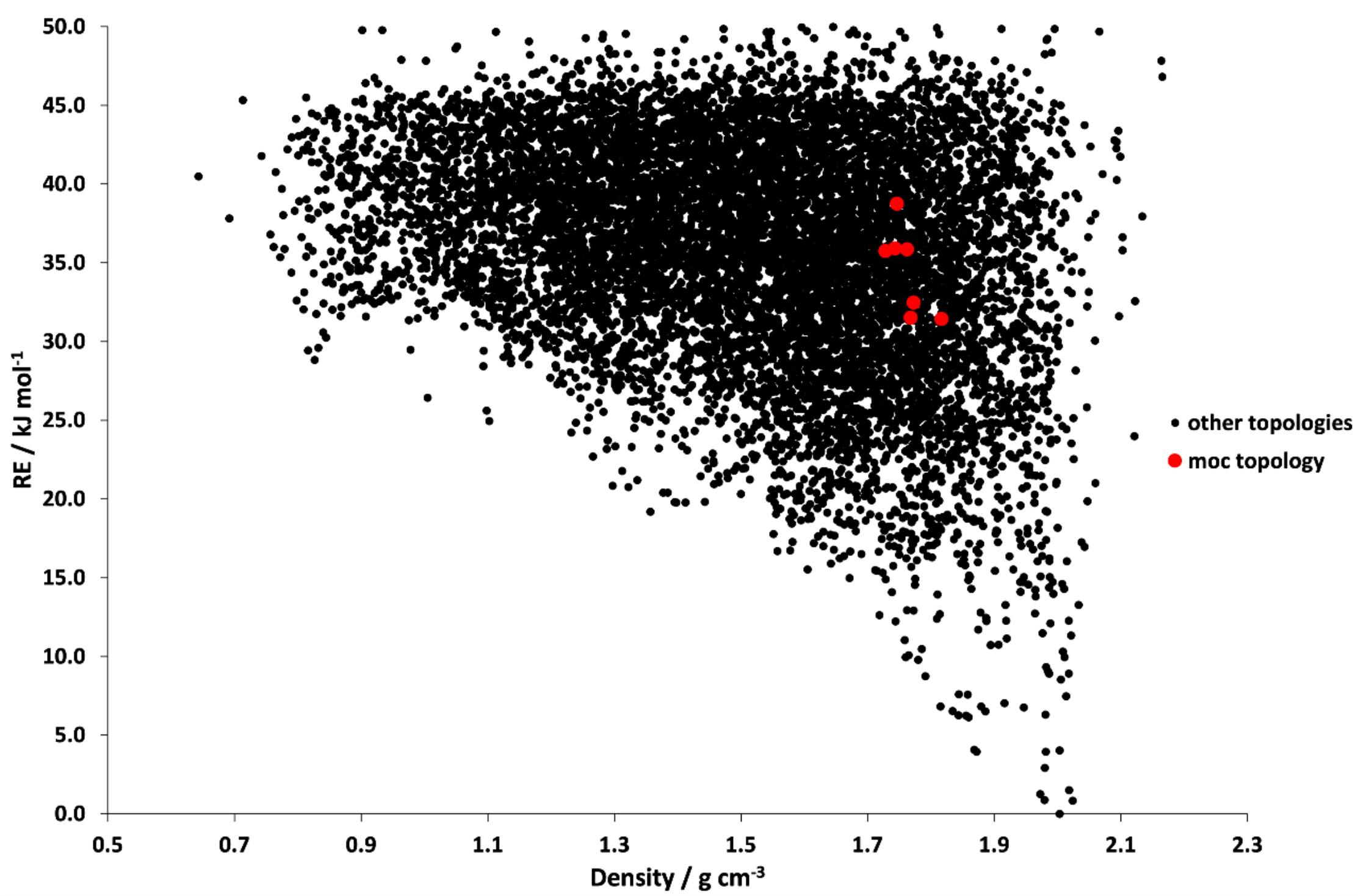

Figure S26. CSP energy landscape highlighting the structures with **moc** topology.

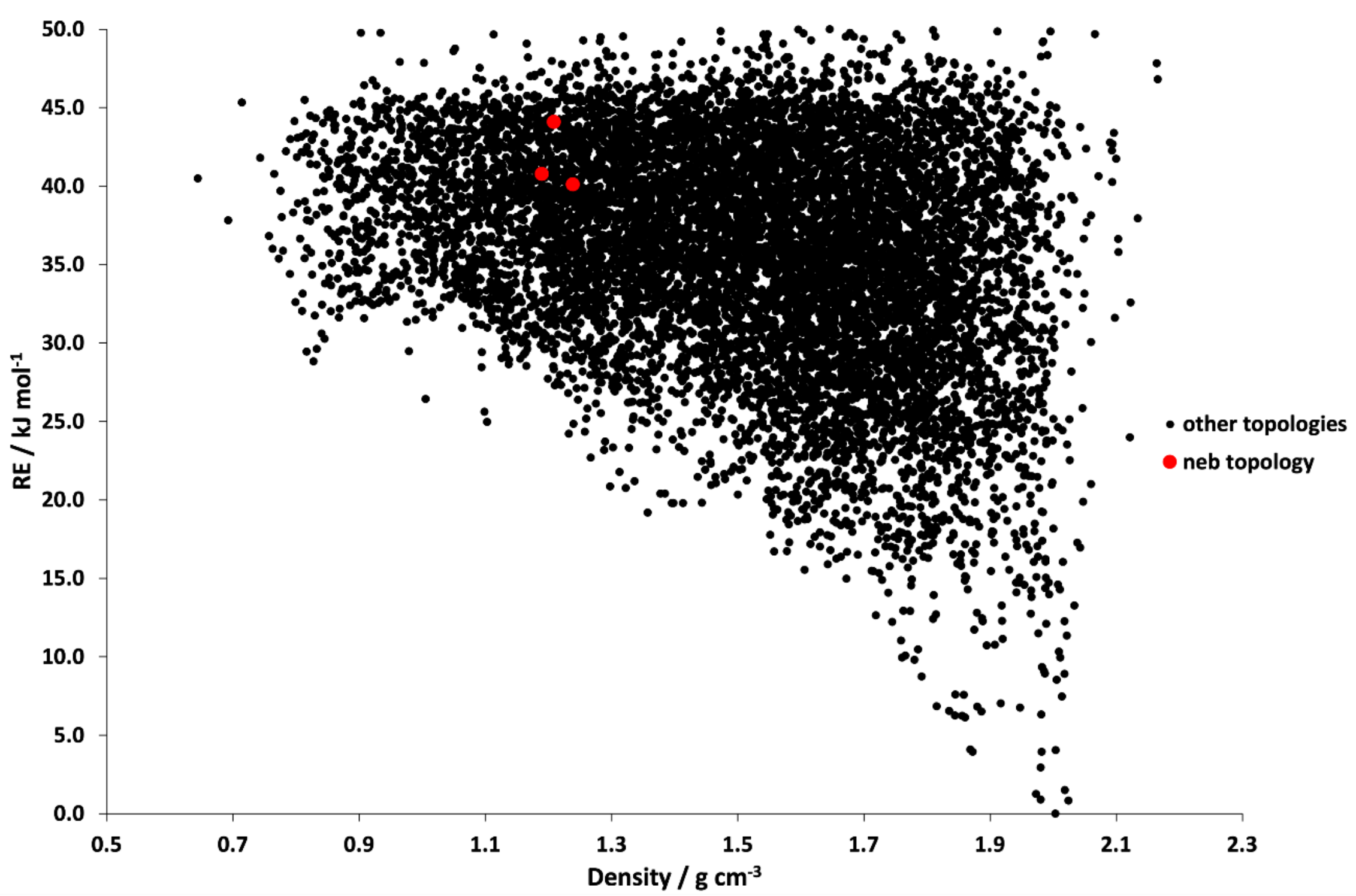

Figure S27. CSP energy landscape highlighting the structures with **neb** topology.

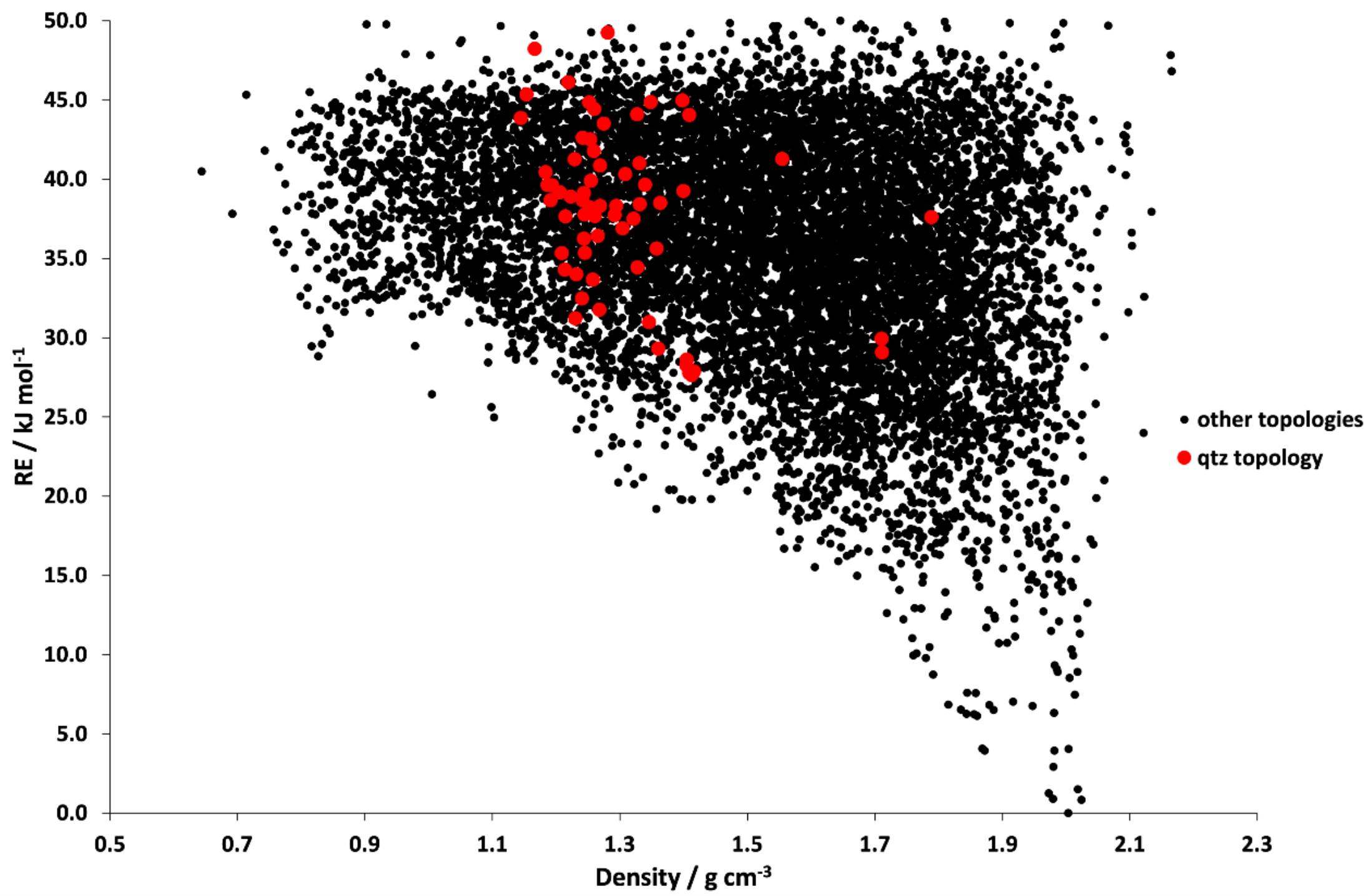

Figure S28. CSP energy landscape highlighting the structures with **qtz** topology

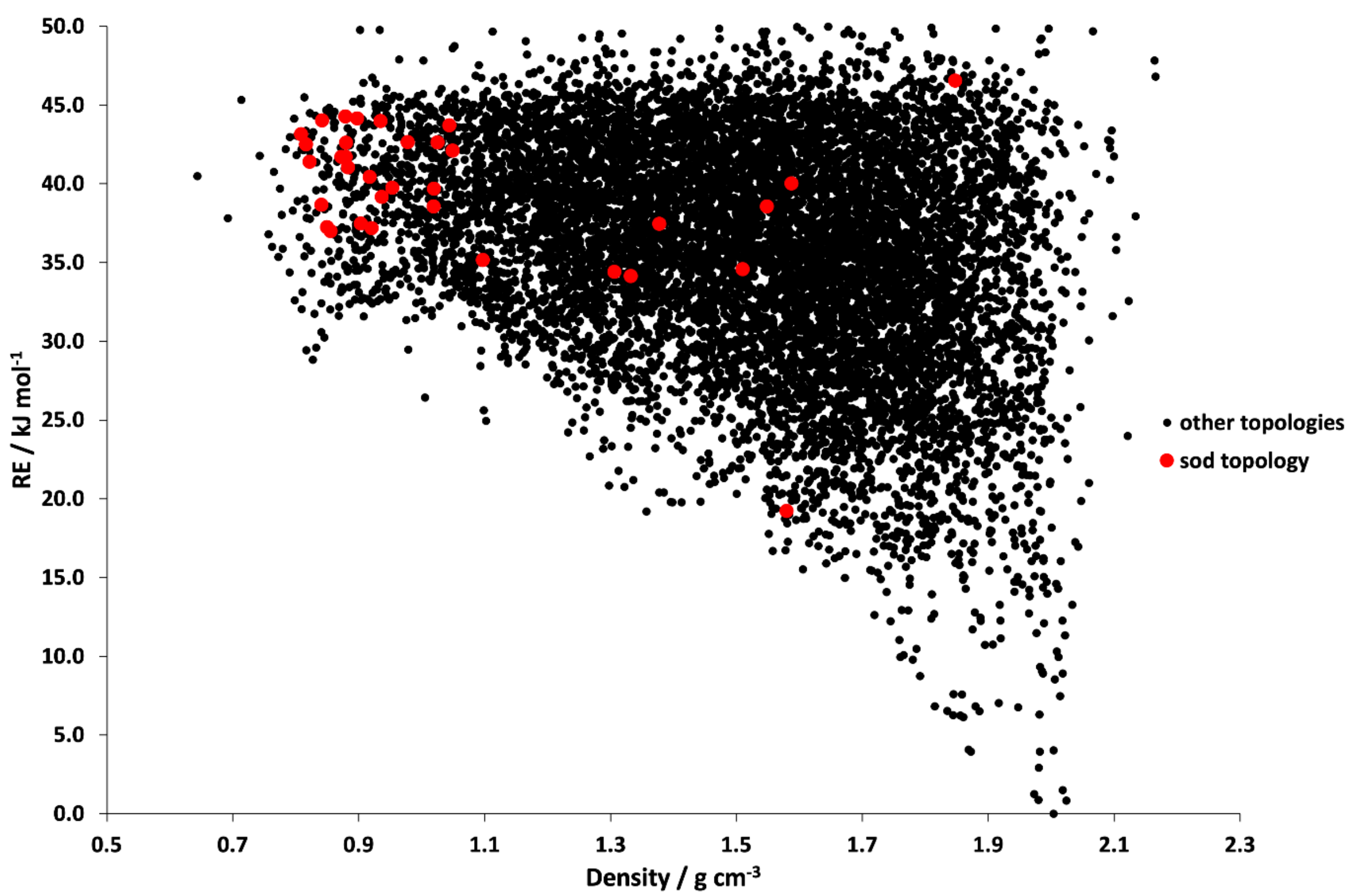

Figure S29. CSP energy landscape highlighting the structures with **sod** topology.

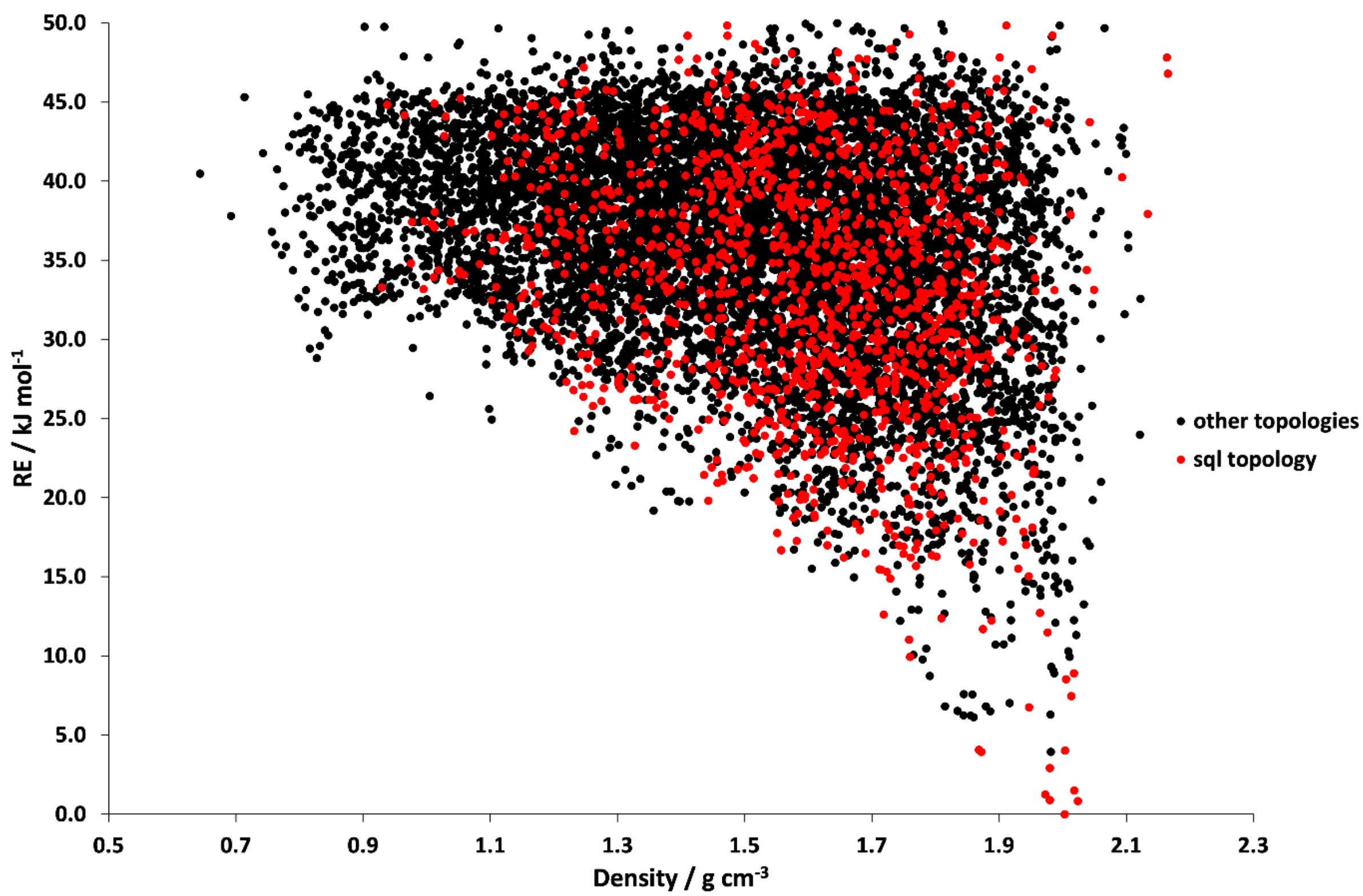

Figure S30. CSP energy landscape highlighting the structures with **sql** topology.

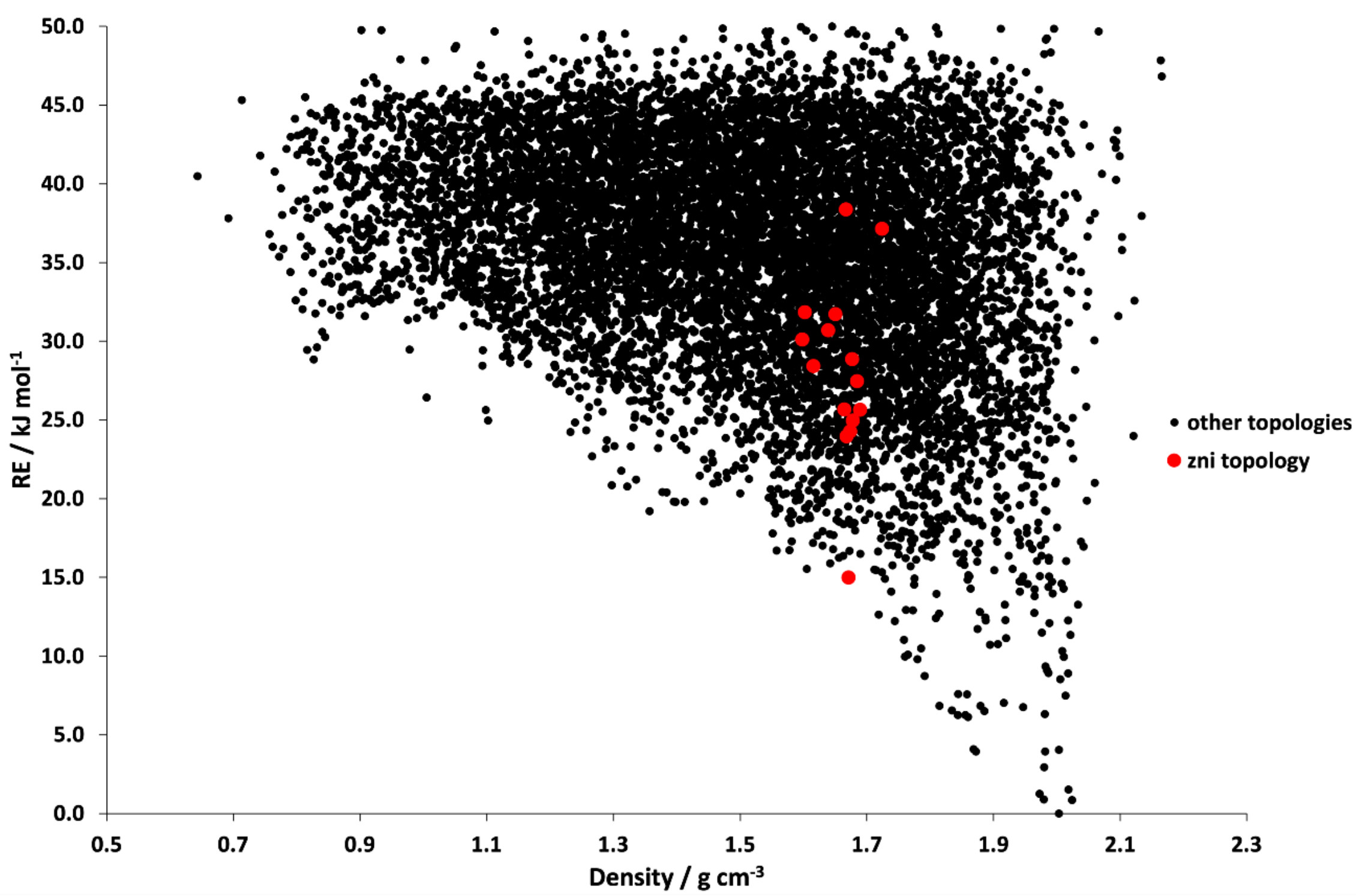

Figure S31. CSP energy landscape highlighting the structures with **zni** topology.

Table S2. Predicted structures identified as full matches to experimentally-observed polymorphs.

Structure name	Energy relative to the global minimum / kJ mol^{-1}	Calculated density / g cm^{-3}	Network topology	CSD match
Znimid2_16_61_Pbca_FRXS3Ki7	31.04	1.137	cag	GIZJOP
Znimid2_8_58_Pnnm_TNVhNp9e	35.00	1.035	crb	GAKXAW
Znimid2_16_94_P42212_3mPlivbU	33.25	0.872	dft	VEJYOZ
Znimid2_8_80_I41_sBzN9sN4	36.00	0.762	gis	EQOCOC01
Znimid2_12_114_P-421c_vYVwKfoC	37.24	0.850	sod	HIFVUO01
Znimid2_16_110_I41cd_JC0of5zc	14.98	1.672	zni	IMIDZB02
Znimid2_8_61_Pbca_3O6xYsHw	17.26	2.038	dia	IMIDZB14

Table S3. Predicted structures identified as partial matches to experimentally-observed polymorphs.

Structure name	Energy relative to the global minimum / kJ mol^{-1}	Calculated density / g cm^{-3}	Network topology	CSD match
Znimid2_12_147_P-3_qO60Y1DX	35.10	0.856	can	PAJRUQ
Znimid2_8_14_P21_c_dDLJx0w7	29.73	1.316	crb	GITTEJ
Znimid2_16_85_P4_n_HgEy04dz	30.20	1.305	crb	VEJYEP
Znimid2_16_56_Pccn_x64k7Fgx	33.97	1.015	crb	VEYJIT
Znimid2_8_23_I222_EAkeZmRJ	35.39	0.813	pcb	ZAVBAD
Znimid2_16_105_P42mc_twl6Oma1	36.81	0.897	atn	USEKIP

S7. Scaling of relative energies with respect to calculated void fraction.

The experimentally-reported structures of Zn(I**m**)$_2$ found in Cambridge structural database (CSD) were geometry-optimized and energy-ranked using the same MLIPs as in the CSP calculation. Analysis of these structures revealed a strong correlation between the calculated energy and void fraction (Figure S19, Table S2). The higher energy polymorphs contain larger solvent-accessible voids, while low energy structures contain smaller voids or are entirely close-packed.

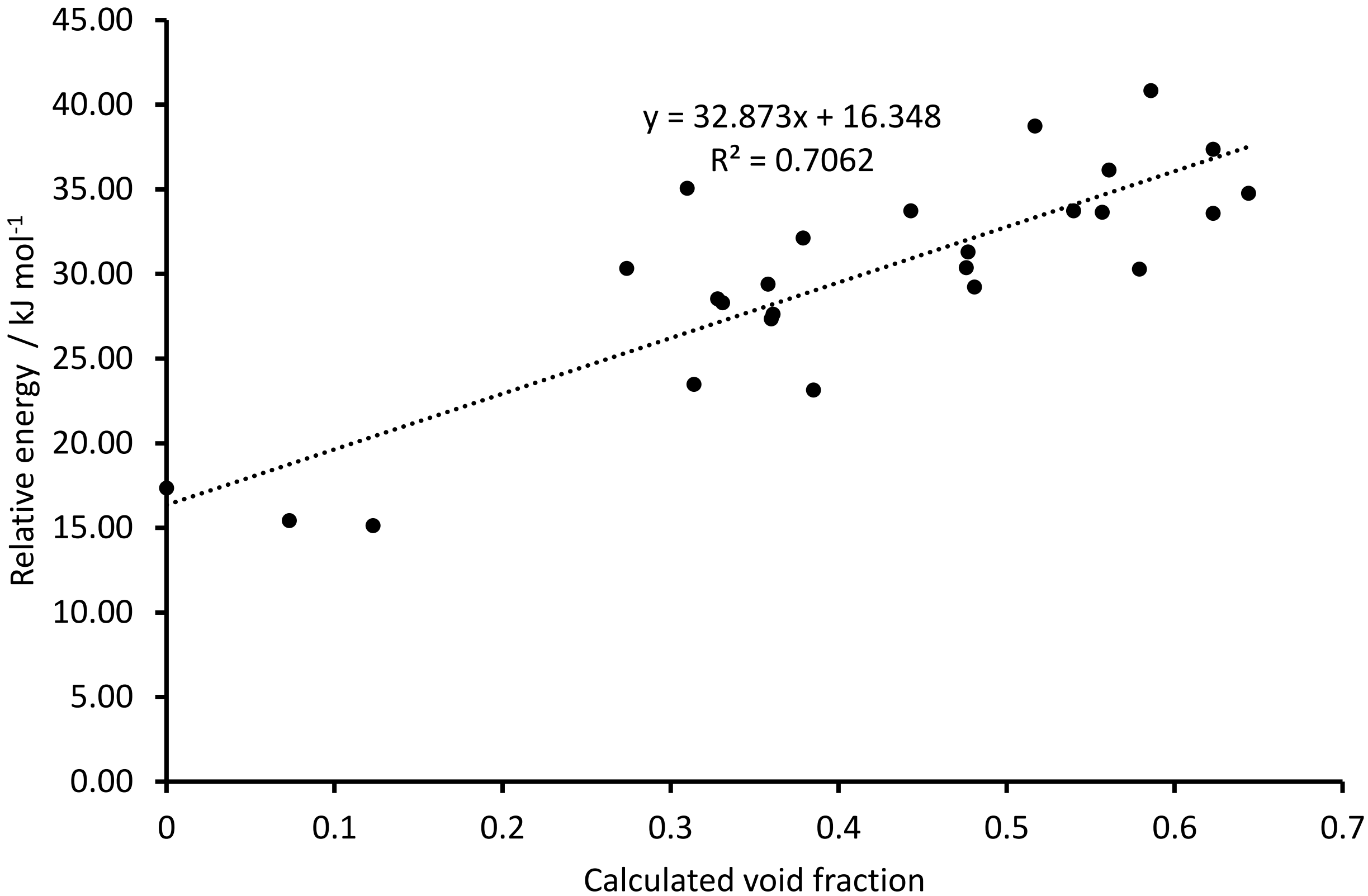

Figure S32. Correlation between the energy and void fraction for the experimentally-reported structures of ZnI**m**$_2$

Table S4. Calculated energies and void fractions for the experimental forms of Zn(I**m**)$_2$.

CSD refcode	Topology	Relative energy, E_{rel} / kJ mol^{-1}	Void fraction, f_{void}	Void-adjusted energy, E' / $kJ\ mol^{-1}$
IMIDZB02	zni	15.12	0.123	11.08
IMIDZB07	coi	15.41	0.073	13.01
IMIDZB14	dia	17.35	0.000	17.35
HIFWAV	nog	23.15	0.385	10.49
GITTEJ	crb	23.47	0.314	13.15
VEJYUF01	cag	27.35	0.36	15.51
GIZJOP	cag	27.62	0.361	15.76
VEJYUF07	cag	28.30	0.331	17.42
GAKXOK	crb	28.53	0.328	17.75
USEKIP	atn	29.22	0.481	13.41
KUDJOK	neb	29.39	0.358	17.62
PAJRUQ	can	30.28	0.579	11.25
VEJYEP	crb	30.32	0.274	21.31
HICGEG	zec	30.36	0.476	14.72
GOQSIQ	10mr	31.30	0.477	15.62
ZAVBUX	hlw1	32.12	0.379	19.66
VEJZIU	mer	33.57	0.623	13.09
VEJYOZ	dft	33.64	0.557	15.33
ZAVBAD	pcb/aco	33.72	0.54	15.97
GAKXAW	crb	33.73	0.443	19.17
DOTCIC	gme	34.77	0.644	13.60
KEVLEE	neb	35.07	0.31	24.88
QOSXUS	aco	36.14	0.561	17.69
EQOCOC01	gis	37.37	0.623	16.89
VEJYIT	crb	38.75	0.517	21.75
HIFVUO	gis	40.83	0.586	21.57
				$E'_{average}$ 16.35±3.66

The energy-porosity relationship found in the experimental polymorphs can be used to assess the synthetic feasibility of the structures generated via CSP. The linear correlation between the lattice energy and void fraction allows us to introduce a void-adjusted energy descriptor under the equation:

$$E' = E_{rel} - k_V \times f_{void} \quad (1)$$

where E_{rel} is the calculated energy of the structure relative to the global minimum; k_V = 32.873 $kJ\ mol^{-1}$ - linear regression coefficient based on the energies and void fractions for the experimental structures; f_{void} is the calculated void fraction.

The least squares analysis of the energies and void fractions of experimental polymorphs of Zn(I**m**)$_2$ gives the values of $E' = (16.348 \pm 3.664)\ kJ\ mol^{-1}$. Assuming the predicted structures follow the same trend as the experimental forms of Zn(I**m**)$_2$ and setting the upper limit of acceptable E' one standard error above the mean value, we obtain a threshold value of

$$E'_{max} = 20.012\ kJ\ mol^{-1}$$

as an upper boundary for the structures that fulfil the synthesizability criterion, leaving 982 structures out of the total 9626 as likely candidates for experimental synthesis.

S8. Comparison of the predicted structures against experimental powder diffraction patterns.

Table S5. Four experimental PXRD patterns from mechanochemical LAG method were selected to verify how the software Critic2 can match experimental PXRD patterns against the predicted CSP structures. The variable-cell similarity score (DIFF) for each pattern is shown, along with the ranking of the matching predicted structure to the experimental pattern.

Structure name and Chemiscope number	Topology	LAG additive	DIFF	Ranking
Znimid2_8_58_Pnnm_TNVhNp9e **3918**	crbT	toluene	0.31	199
Znimid2_16_110_I41cd_JC0of5zc **75**	zni	methanol	0.02	6
Znimid2_16_61_Pbca_FRXS3Ki7 **2263**	cag	dimethylformamide	0.21	473
Znimid2_16_61_Pbca_FRXS3Ki7 **2263**	cag	choloform	0.23	763

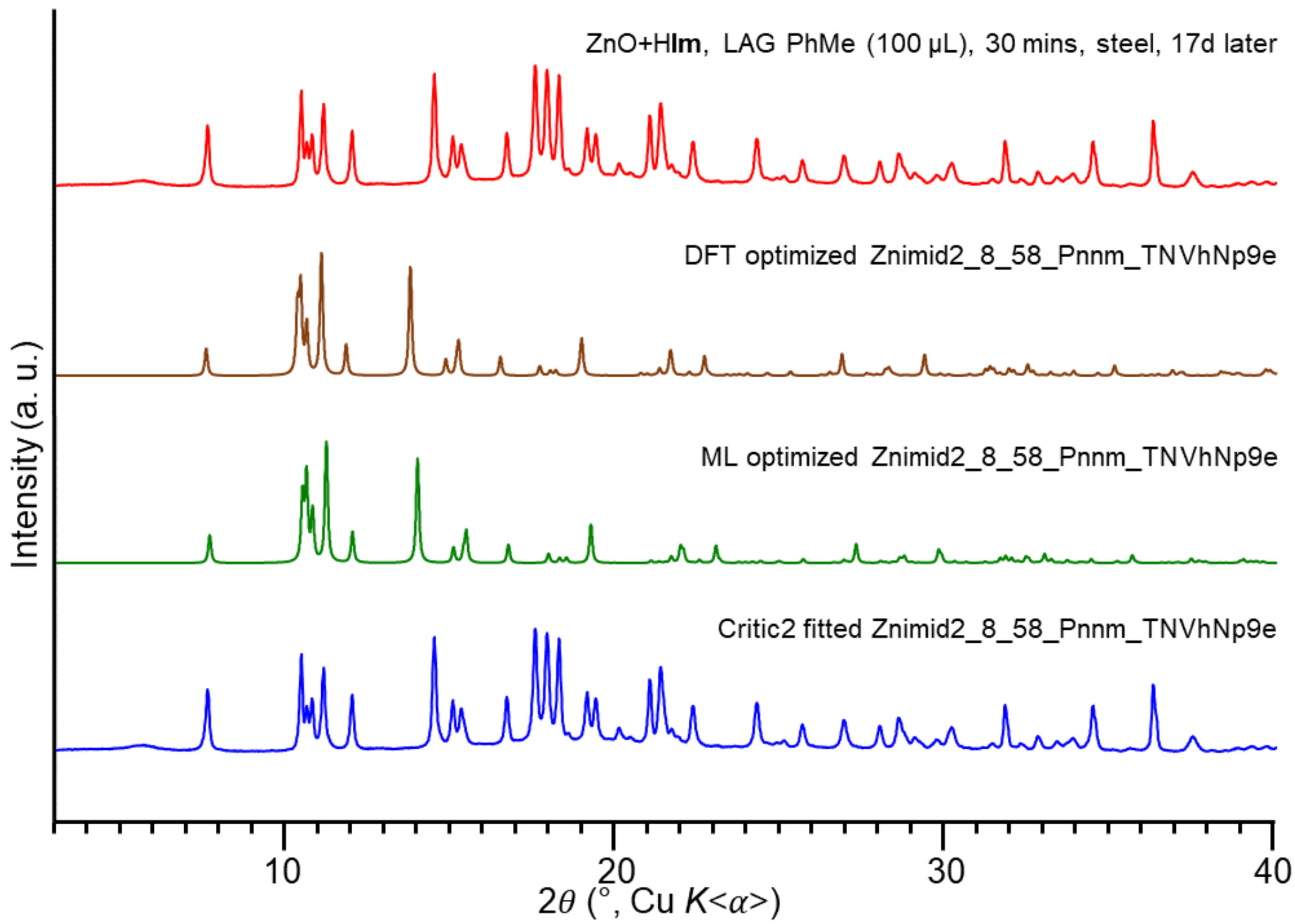

Figure S33. PXRD patterns overall from top to bottom for: experimentally measured pattern for Zn(**Im**)$_2$ with **crbT** (CSD GAKXAW) topology (red); simulated DFT optimized predicting structure pattern (brown); simulated pattern from ML optimized structure (green); Critic2 fitted PXRD pattern from the ML-optimized structure (blue).

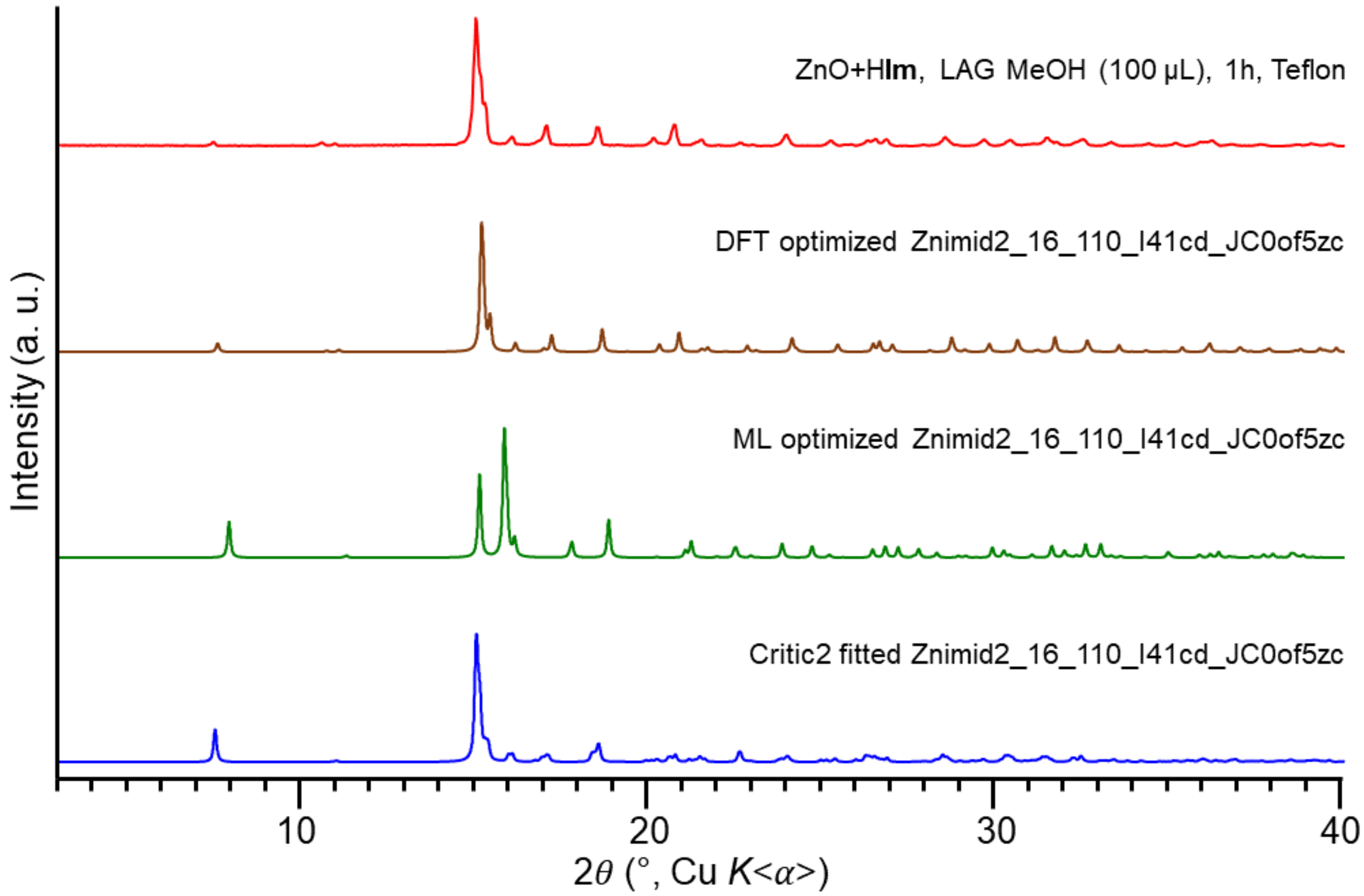

Figure S34. PXRD patterns overall from top to bottom for: experimentally measured pattern for Zn(**Im**)$_2$ with **zni** (CSD IMIDZB02) topology (red); simulated DFT optimized predicting structure pattern (brown); simulated pattern from ML optimized structure (green); Critic2 fitted PXRD pattern from the ML-optimized structure (blue).

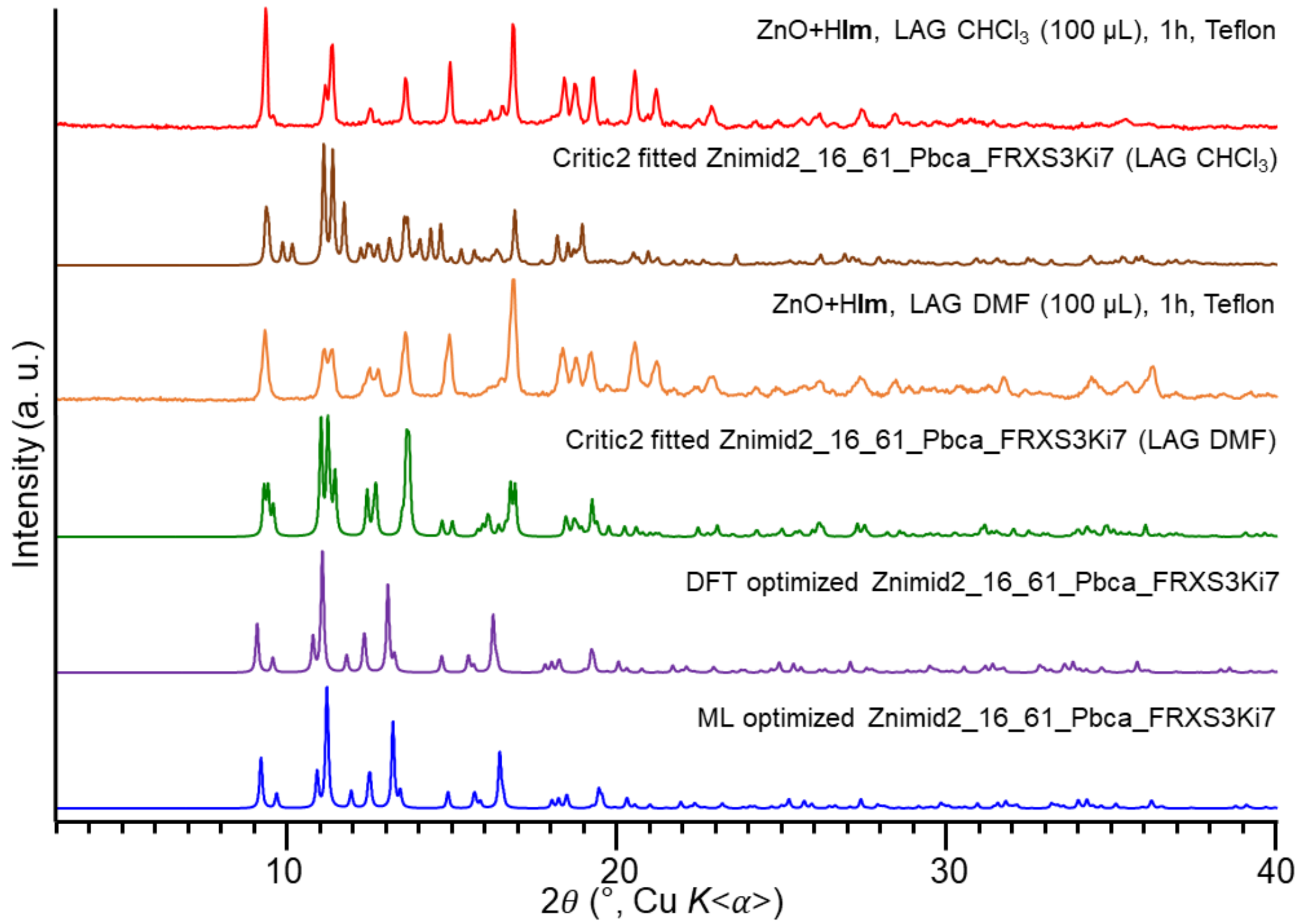

Figure S35. PXRD patterns overall from top to bottom for: experimentally measured pattern, using chloroform as LAG, for Zn(**Im**)$_2$ with **cag** (CSD GAKXEA) topology (red);); Critic2 fitted PXRD pattern with chloroform as LAG (brown); experimentally measured PXRD pattern using DMF as LAG (orange); Critic2 fitted PXRD pattern with chloroform as LAG (green); simulated DFT optimized predicting structure pattern (purple); simulated pattern from ML optimized structure (blue).

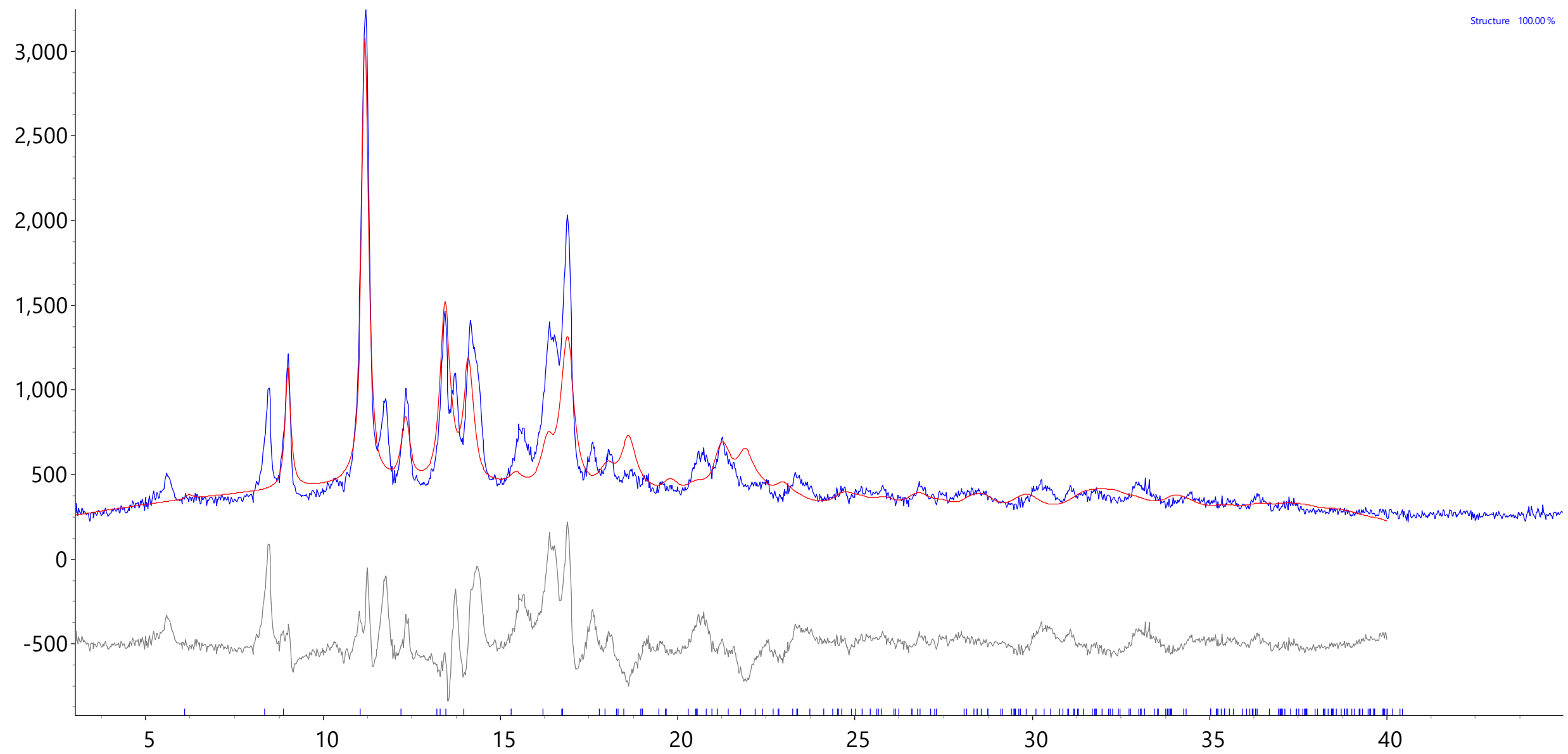

Figure S36. Rietveld refinement plot for the best matching predicted structure for the material obtained by heating **crb-**$ZnIm_2$ at 150 °C for 3 hours. The structure identifier is Znimid2_16_56_Pccn_YMQOEPiX, located in the Chemiscope file under the number 9563. The experimental profile is shown in blue, the calculated profile is shown in red, and the difference curve is shown in grey. Evidently there are significant discrepancies between the experimental and calculated diffraction profile, indicating that the current structural model requires more work before it can be counted as a fully-accurate structure determination.

S9. References

(1) Pickard, C. J.; Needs, R. J. *Ab Initio* Random Structure Searching. *J. Phys. Condens. Matter* **2011**, *23* (5), 053201. https://doi.org/10.1088/0953-8984/23/5/053201.

(2) Darby, J. P.; Arhangelskis, M.; Katsenis, A. D.; Marrett, J. M.; Friščić, T.; Morris, A. J. *Ab Initio* Prediction of Metal-Organic Framework Structures. *Chem. Mater.* **2020**, *32* (13), 5835–5844. https://doi.org/10.1021/acs.chemmater.0c01737.

(1) Clark, S. J.; Segall, M. D.; Pickard, C. J.; Hasnip, P. J.; Probert, M. I. J.; Refson, K.; Payne, M. C., *Zeitschrift für Krist-Cryst. Mater.*, **2005**, *220* (5–6), 567–570.

(4) Monkhorst, H. J.; Pack, J. D. Special Points for Brillouin-Zone Integrations. *Phys. Rev. B* **1976**, *13* (12), 5188–5192. https://doi.org/10.1103/PhysRevB.13.5188.

(5) Schütt, K. T.; Kessel, P.; Gastegger, M.; Nicoli, K. A.; Tkatchenko, A.; Müller, K.-R. SchNetPack: A Deep Learning Toolbox For Atomistic Systems. *J. Chem. Theory Comput.* **2019**, *15* (1), 448–455. https://doi.org/10.1021/acs.jctc.8b00908.

(6) Schütt, K. T.; Unke, O. T.; Gastegger, M. Equivariant Message Passing for the Prediction of Tensorial Properties and Molecular Spectra. arXiv June 7, 2021. https://doi.org/10.48550/arXiv.2102.03150.

(7) Hjorth Larsen, A.; Jørgen Mortensen, J.; Blomqvist, J.; Castelli, I. E.; Christensen, R.; Dułak, M.; Friis, J.; Groves, M. N.; Hammer, B.; Hargus, C.; Hermes, E. D.; Jennings, P. C.; Bjerre Jensen, P.; Kermode, J.; Kitchin, J. R.; Leonhard Kolsbjerg, E.; Kubal, J.; Kaasbjerg, K.; Lysgaard, S.; Bergmann Maronsson, J.; Maxson, T.; Olsen, T.; Pastewka, L.; Peterson, A.; Rostgaard, C.; Schiøtz, J.; Schütt, O.; Strange, M.; Thygesen, K. S.; Vegge, T.; Vilhelmsen, L.; Walter, M.; Zeng, Z.; Jacobsen, K. W. The Atomic Simulation Environment—a Python Library for Working with Atoms. *J. Phys. Condens. Matter* **2017**, *29* (27), 273002. https://doi.org/10.1088/1361-648X/aa680e.

(8) Kingma, D. P.; Ba, J. Adam: A Method for Stochastic Optimization. arXiv January 29, 2017. http://arxiv.org/abs/1412.6980 (accessed 2024-05-17).

(9) Otero-de-la-Roza, A.; Blanco, M. A.; Pendás, A. M.; Luaña, V. Critic: A New Program for the Topological Analysis of Solid-State Electron Densities. *Comput. Phys. Commun.* **2009**, *180* (1), 157–166. https://doi.org/10.1016/j.cpc.2008.07.018.

(10) Otero-de-la-Roza, A.; Johnson, E. R.; Luaña, V. Critic2: A Program for Real-Space Analysis of Quantum Chemical Interactions in Solids. *Comput. Phys. Commun.* **2014**, *185* (3), 1007–1018. https://doi.org/10.1016/j.cpc.2013.10.026.

(11) Spek, A. L. Single-Crystal Structure Validation with the Program *PLATON*. *J. Appl. Crystallogr.* **2003**, *36* (1), 7–13. https://doi.org/10.1107/S0021889802022112.

(12) Sykes, R. A.; Johnson, N. T.; Kingsbury, C. J.; Harter, J.; Maloney, A. G. P.; Sugden, I. J.; Ward, S. C.; Bruno, I. J.; Adcock, S. A.; Wood, P. A.; McCabe, P.; Moldovan, A. A.; Atkinson, F.; Giangreco, I.; Cole, J. C. What Has Scripting Ever Done for Us? The CSD Python Application Programming Interface (API). *J. Appl. Crystallogr.* **2024**, *57* (4), 1235–1250. https://doi.org/10.1107/S1600576724005934.

(13) Blatov, V. A.; Shevchenko, A. P.; Proserpio, D. M. Applied Topological Analysis of Crystal Structures with the Program Package Topospro. *Cryst. Growth Des.* **2014**, *14* (7), 3576–3586. https://doi.org/10.1021/cg500498k.

(14) Bruno, I. J.; Cole, J. C.; Edgington, P. R.; Kessler, M.; Macrae, C. F.; McCabe, P.; Pearson, J.; Taylor, R. New Software for Searching the Cambridge Structural Database and Visualizing Crystal Structures. *Acta Crystallogr. B* **2002**, *58* (3), 389–397. https://doi.org/10.1107/S0108768102003324.

(15) Grimme, S.; Antony, J.; Ehrlich, S.; Krieg, H. A Consistent and Accurate *Ab Initio* Parametrization of Density Functional Dispersion Correction (DFT-D) for the 94 Elements H-Pu. *J. Chem. Phys.* **2010**, *132* (15), 154104. https://doi.org/10.1063/1.3382344.

(16) Batatia, I.; Kovacs, D. P.; Simm, G.; Ortner, C.; Csanyi, G. MACE: Higher Order Equivariant Message Passing Neural Networks for Fast and Accurate Force Fields. In *Advances in Neural Information Processing Systems*; Koyejo, S., Mohamed, S., Agarwal, A., Belgrave, D., Cho, K., Oh, A., Eds.; Curran Associates, Inc., 2022; Vol. 35, pp 11423–11436.

(17) Batatia, I.; Benner, P.; Chiang, Y.; Elena, A. M.; Kovács, D. P.; Riebesell, J.; Advincula, X. R.; Asta, M.; Avaylon, M.; Baldwin, W. J.; Berger, F.; Bernstein, N.; Bhowmik, A.; Bigi, F.; Blau, S. M.; Cărare, V.; Ceriotti, M.; Chong, S.; Darby, J. P.; De, S.; Della Pia, F.; Deringer, V. L.; Elijošius, R.; El-Machachi, Z.; Fako, E.; Falcioni, F.; Ferrari, A. C.; Gardner, J. L. A.; Gawkowski, M. J.; Genreith-Schriever, A.; George, J.; Goodall, R. E. A.; Grandel, J.; Grey, C. P.; Grigorev, P.; Han, S.; Handley, W.; Heenen, H. H.; Hermansson, K.; Ho, C. H.; Hofmann, S.; Holm, C.; Jaafar, J.; Jakob, K. S.; Jung, H.; Kapil, V.; Kaplan, A. D.; Karimitari, N.; Kermode, J. R.; Kourtis, P.; Kroupa, N.; Kullgren, J.; Kuner, M. C.; Kuryla, D.; Liepuoniute, G.; Lin, C.; Margraf, J. T.; Magdău, I.-B.; Michaelides, A.; Moore, J. H.; Naik, A. A.; Niblett, S. P.; Norwood, S. W.; O'Neill, N.; Ortner, C.; Persson, K. A.; Reuter, K.; Rosen, A. S.; Rosset, L. A. M.; Schaaf, L. L.; Schran, C.; Shi, B. X.; Sivonxay, E.; Stenczel, T. K.; Sutton, C.; Svahn, V.; Swinburne, T. D.; Tilly, J.; Van Der Oord, C.; Vargas, S.; Varga-Umbrich, E.; Vegge, T.; Vondrák, M.; Wang, Y.; Witt, W. C.; Wolf, T.; Zills, F.; Csányi, G. A Foundation Model for Atomistic Materials Chemistry. *J. Chem. Phys.* **2025**, *163* (18), 184110. https://doi.org/10.1063/5.0297006.

(18) Brekalo, I.; Lisac, K.; Ramirez, J. R.; Pongrac, P.; Puškarić, A.; Valić, S.; Xu, Y.; Ferguson, M.; Marrett, J. M.; Arhangelskis, M.; Friščić, T.; Holman, K. T. Mechanochemical Solid Form Screening of Zeolitic Imidazolate Frameworks Using Structure-Directing Liquid Additives. *J. Am. Chem. Soc.* **2025**, *147* (31), 27413–27430. https://doi.org/10.1021/jacs.5c04043.

(19) Rietveld, H. M. A Profile Refinement Method for Nuclear and Magnetic Structures. *J. Appl. Crystallogr.* **1969**, *2* (2), 65–71. https://doi.org/10.1107/S0021889869006558.

(20) Coelho, A. A. TOPAS and TOPAS-Academic : An Optimization Program Integrating Computer Algebra and Crystallographic Objects Written in C++. *J. Appl. Crystallogr.* **2018**, *51* (1), 210–218. https://doi.org/10.1107/S1600576718000183.
